# Nearby Sites Show Similar Upwind Sources and Differing Semivolatile Concentrations in Coastal Aerosol Particles

**DOI:** 10.1021/acsestair.5c00191

**Published:** 2025-11-22

**Authors:** Sanghee Han, Abigail S. Williams, Lynn M. Russell, Veronica Z. Berta, Jeramy L. Dedrick, Christian Pelayo, Nattamon Maneenoi, Atsushi Osawa, Israel Silber, Damao Zhang, Maria A. Zawadowicz, Arthur J. Sedlacek

**Affiliations:** † Scripps Institution of Oceanography, 70015 University of California San Diego, San Diego, California 92093, United States; ‡ Pacific Northwest National Laboratory, Richland, Washington 99352, United States; § Brookhaven National Laboratory, Upton, New York 11973, United States

**Keywords:** Coastal aerosol, Land−sea breeze, Local traffic, Upwind sources, Semivolatile components

## Abstract

The Eastern Pacific Cloud Aerosol Precipitation Experiment (EPCAPE) characterized aerosol composition using measurements at two sites within 3 km (Scripps Pier and Mt. Soledad) from 15 February 2023 to 14 February 2024. Comparing the two sites shows the strong influence of upwind sources that results in similar monthly compositions at both sites. The seasonal changes in chemical mass concentrations were largely driven by the upwind source regions, with coastal northwesterly back-trajectories occurring 63–65% of the year and bringing submicrometer mass concentrations that were lower than the EPCAPE average for all trajectories at each site. In contrast, refractory black carbon (rBC) and nonrefractory (NR)-organics and nitrate mass concentrations exceeded EPCAPE average concentrations for back-trajectories from urban areas such as Los Angeles-Long Beach. For hourly measurements, NR-organics and non-sea-salt (NSS)-sulfate mass concentrations at Mt. Soledad were correlated strongly (*r* = 0.73–0.82) to those measured at Scripps Pier, but NR-nitrate was correlated only moderately (*r* = 0.63). The explanation for the lower correlation of NR-nitrate is both emissions between the sites and semivolatility, with semivolatility accounting for site-to-site changes in daily averages of +0.01 μg m^–3^ per percentage site-to-site difference in relative humidity and −0.07 μg m^–3^ per degree Celsius site-to-site difference in temperature. On average, comparing Scripps Pier to Mt. Soledad, NR-nitrate was higher by 29% because of relative humidity and lower by −26% because of temperature. NR-nitrate and rBC mass concentrations at Scripps Pier for nighttime were 13–15% higher than those for daytime because land breezes brought higher inland concentrations. Concentrations of rBC were 52% higher at Mt. Soledad than those measured at Scripps Pier, accompanied by increases in tracers for brake wear because of traffic on the steep roads within 10 m of that site. The implications are that these nearby sites had comparable monthly concentrations of measured components due to their similar back-trajectories, but hourly and daily concentration differences supported quantification of the meteorological effects from relative humidity and temperature on semivolatile NR-nitrate as well as minor differences from land–sea breezes and local emissions.

## Graphical abstract

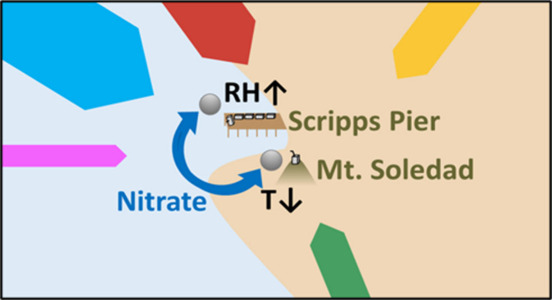

## 1 Introduction

Characterizing the chemical composition of submicron particles (PM_1_, particulate matter with an aerodynamic diameter less than 1 μm) is important for understanding their impact on both human health^1–3^ and climate.^4^ Coastal regions often experience a complex mixture of atmospheric aerosols from marine and continental sources depending on whether back-trajectories come from offshore or inland regions. Inland regions include anthropogenic emissions of nitrate, ammonium, and refractory black carbon (rBC) from local and regional urban areas,^5–7^ and offshore trajectories bring natural aerosols of sea salt and sulfate from the ocean.^8–10^ Mineral dust can also be transported from agricultural or arid inland areas to contribute to the aerosol composition.^11,12^ Perhaps the largest effect on coastal aerosols is the influence of long-range transport of pollutants from urban regions that are located directly upwind.^13,14^ This variety of sources, combined with the limited availability of observations, contributes to uncertainties in the representation of aerosol composition in air quality and climate prediction.^15–17^ The Eastern Pacific Cloud Aerosol Precipitation Experiment (EPCAPE) was designed to investigate an example of the complex contributions to coastal aerosols by measuring them for 12 months at two nearby sites in southern California while characterizing the local meteorology at the sites.

Along the southern California coast, prevailing surface winds often pass slightly offshore of the coastline from the northwesterly direction. Occasionally these winds pass over land, transporting aerosol from the Los Angeles-Long Beach urban port area, which has been shown to contribute to organic composition^18,19^ as well as to soot, sulfate, and nitrate.^20,21^ Easterly winds occur in winter months, with Day et al. using potential source contribution functions (PSCF) to show that nitrate emissions are often from urban sources in the vicinity of Riverside.^22^ Southerly trajectories have transported refractory black carbon (rBC) from Tijuana into San Diego.^23^ A quantitative apportionment of contributions from these upwind regions near San Diego to aerosol chemical compositions throughout the year is needed before local differences can be assessed.

In addition to the contributions of source regions, coastal sites can also have steep gradients in temperature and relative humidity (RH) across very short distances (∼1 km), which lead to differences in the partitioning of semivolatile components between the gas and particle phases.^24^ This partitioning of semivolatile components has been demonstrated in laboratory measurements for ammonium and nitrate, inter alia,^25,26^ and has been investigated with thermodynamic models of several semivolatile components,^27–29^ but there are few direct observations of this effect in the atmosphere. For example, Li et al. used simulations to show that temperature and RH significantly influence the distribution of nitrate aerosol in China with the ISORROPIA-II thermodynamic model.^27^ ISORROPIA-II was also used by Guo et al. to show that temperature and RH strongly influence the particle pH, which in turn affects the partitioning between the gas and particle phases of inorganic components like ammonium, nitrate, and chloride.^28^ Air quality model simulations clearly illustrate the partitioning of ammonium and nitrate in urban regions,^30^ but few observations provide quantitative constraints on this effect in ambient conditions. Observational constraints are needed because the magnitude of RH and temperature effects on semivolatile compounds depends on the specific properties of mixtures present in aerosol particles (which determine their acidity), and these mixture properties are generally not measured directly and have simplified representations in models.

Coastal meteorology is often characterized by a diurnal pattern of land–sea breezes, which transport aerosols from offshore during the day and from inland at night.^31–35^ For example, land breezes bring inland aerosols, often including urban emissions, to offshore regions of Taiwan and cause high PM concentrations at night.^34^ The chemical composition of nonrefractory (NR)-PM_1_ has been shown to vary significantly between periods dominated by sea breezes and those influenced by land breezes in a coastal city of southeast China.^33^ This land–sea breeze effect has been observed in the southern California region,^36,37^ with consequences for air quality.^38,39^ However, land–sea breezes change aerosol composition only for components with concentration gradients near the coast, as concentrations that are similar offshore and inland will change negligibly relative to upwind sources when wind shifts from land to sea breeze.

Local traffic sources contribute pollutants related to vehicle exhaust and nonexhaust emissions.^40^ Exhaust emissions from fuel combustion include rBC and NO_*x*_,^41,42^ while nonexhaust emissions, such as the mechanical abrasion of tires^43^ and brakes,^44^ can also contribute to local aerosol composition.^45,46^ These traffic emissions have contributed to diurnal patterns in aerosol levels with the land–sea breeze in coastal areas.^47^ However, the magnitude of this effect depends not only on the gradients in emissions near the coast but also on the time scale of formation for different components, and these time scales are different for southern California compared to other coastal regions.

This study compares the two coastal sites (Scripps Pier and Mt. Soledad in La Jolla, CA, USA) that were used for measuring aerosol chemical composition during EPCAPE.^48^ We identify the frequency of different upwind sources, showing that these sources explain most of the seasonal changes in aerosol composition over the year and account for the similarities in the monthly compositions between the sites. We then investigate the differences between the sites on time scales of hours to days. Despite the proximity of the sites, there are daily differences in the temperature and relative humidity that affect the partitioning of semivolatile inorganic components. In addition, morning and evening transitions in land–sea breezes result in night–day differences at both sites, with the offshore location of Scripps Pier having a larger concentration difference than Mt. Soledad. Other differences between the sites are associated with the local sources that contribute emissions between and surrounding the two sites.

## 2 Methods

EPCAPE was conducted from 15 February 2023 to 14 February 2024 by the U.S. Department of Energy Atmospheric Radiation Measurement (ARM) facility. The main measurement site was stationed at the Ellen Browning Scripps Memorial Pier (hereafter, “Scripps Pier”; 32.887° N, −117.257° E; 330 m offshore and 18 m ASL) in La Jolla, CA, USA. Additional instruments were deployed at a secondary site at Mt. Soledad (32.840° N, −117.249° E; 3 km southeast of Scripps Pier and 250 m ASL). At Scripps Pier, aerosol measurements were provided by instrumentation housed in ARM’s Aerosol Observing System (AOS). At Mt. Soledad, aerosol measurements were housed in an aerosol sampling container using an isokinetic, wind-facing inlet.^49^ Aerosol measurements with aerosol particle concentrations exceeding 8000 cm^–3^ were excluded to remove “spikes” in composition from sporadic, short contamination events at the sampling sites, which accounted for 2% of measurements at Mt. Soledad and 13% at Scripps Pier. To compare conditions that were similar at both sites, measurements at times with clouds at Mt. Soledad (13% of EPCAPE based on measured visibility <5 km) and with precipitation at either site (5% of EPCAPE with rain rate >0 mm/h at Scripps Pier) were excluded (Figure S1). Throughout this work, the correlation coefficients (*r*) are Pearson and are described as weak (0.1 ≤ *r* < 0.3), moderate (0.3 ≤ *r* < 0.7), and strong (0.7 ≤ *r*).^50^

### 2.1 Online Aerosol and Trace Gas Instrumentation

At Scripps Pier, an Aerodyne Aerosol Chemical Speciation Monitor (ACSM; Aerodyne Research Inc., Billerica, MA, USA) provides 30 min average mass concentrations of nonrefractory (NR) organic, sulfate, nitrate, ammonium, and chloride mass fragments in PM_1_ particles (effectively sampling 40–800 nm in diameter).^51,52^ A single-particle soot photometer (SP2) monitored the rBC mass concentration every 1 min by utilizing incandescence signals produced by black carbon-containing particles 75–500 nm in diameter.^53^ The concentrations of absorption-equivalent black carbon (eBC) in submicron dry aerosols were measured using an Aethalometer by optical attenuation.^54^ For black carbon, eBC mass concentrations generally follow the seasonal differences observed for rBC (Figure S3), although eBC concentrations were consistently about 8 times higher than rBC concentrations. This difference between eBC and rBC is likely explained by the uncertainties in the mass absorption cross section (MAC) and correction factor, which are applied to convert absorption to black carbon mass loading for eBC, as well as by the incidental inclusion of dark brown carbon^63,64^ or dust particles^65^ in the eBC measurement. Dry particle number size distributions were measured by a scanning mobility particle sizer (SMPS; TSI Incorporated)^55^ and aerodynamic particle sizer (APS; TSI Incorporated).^56^ The carbon monoxide (CO) mixing ratio was measured by a CO analyzer,^57^ and ozone (O_3_) concentration was measured by an O_3_ analyzer.^58^ Minor instrument outages are documented by DOE ARM.^59,60^

At Mt. Soledad, a high-resolution time-of-flight aerosol mass spectrometer (HR-ToF-AMS; Aerodyne Research Inc.) measured the size-resolved NR mass concentration every 5 min. A single-particle soot photometer (SP2; Droplet Measurement Technologies) measured the rBC mass concentration, and a scanning electrical mobility spectrometer (SEMS; Brechtel Mfg. Inc.) and aerodynamic particle sizer (APS; TSI) measured the dry aerosol number size distribution. The operation and calibration of the SP2 and SEMS measurements are described in work by Betha et al.^61^ Ambient O_3_ concentration was measured by a UV photometer-based O_3_ analyzer (Thermo 49C). Instrument outages are documented in the posted data set^62^ and include two significant gaps for instrument repairs: HR-ToF-AMS measurements were not available from 24 December 2023 through 25 January 2024, and SP2 measurements were not available from 1 March 2023 to 3 May 2023. Other minor instrument outages are documented with the posted data set.^62^

### 2.2 X-ray Fluorescence of Daily Submicron Filters

In addition to the online aerosol measurements at Scripps Pier and Mt. Soledad, filter samples were collected each day on prescanned Teflon filters (Teflon, Pall Inc., 37 mm diameter, 1.0 μm pore size) behind the sharp-cut cyclones (SCC2.229 PM_1_, BGI Inc.) at both sites from 7 pm (local time) to 6 pm the following day with a sample flow of 10 LPM. Samples were transported to the laboratory and stored frozen. To provide the two-site weekly average concentrations of refractory dust and sea salt, filters collected on Wednesdays and Saturdays at Scripps Pier and on Sundays at Mt. Soledad were analyzed by Chester Labnet (Tigard, OR, USA) with X-ray fluorescence (XRF) analysis to give the mass concentrations of elements, including Na, Mg, Al, Si, P, S, Cl, K, Ca, Ti, V, Cr, Mn, Fe, Co, Ni, Cu, Zn, Br, Rb, Sr, Zr, Ag, Pb, and Ba. Sea salt mass concentrations were determined as Na (μg m^–3^) × 1.47 + Cl (μg m^–3^).^12,66,67^ The mass of dust was calculated from XRF metal concentrations, assuming dust consists of MgCO_3_, Al_2_O_3_, SiO_2_, K_2_O, CaCO_3_, TiO_2_, Fe_2_O_3_, MnO, and BaO after excluding the mass associated with sea salt.^12,68,69^ Sea salt and dust concentrations were calculated as weekly PM_1_ averages of the Wednesday, Saturday, and Sunday filters.

### 2.3 Aerosol Composition Corrections

The components measured by both HR-ToF-AMS and ACSM are referred to as nonrefractory (NR) and are defined as fragments that were ionized by electron impact after vaporizing on an ∼600 °C surface. The mass concentrations and size distribution of NR mass from both HR-ToF-AMS and ACSM were corrected using a monthly average collection efficiency (CE). This CE value was derived based on comparisons of HR-ToF-AMS and particle size distribution-derived mass concentrations after the subtraction of 23 h average refractory component (rBC, sea salt, and dust) mass. Size distributions from SEMS (0.011–0.90 μm) or SMPS (0.011–0.50 μm) were merged with those from APS (0.50–10 μm) to calculate submicron mass concentration.^70^

Non-sea-salt (NSS)-sulfate and NSS-chloride concentrations were calculated from NR-sulfate and NR-chloride by subtracting sea salt (SS)-sulfate and SS-chloride derived from the SS mass concentration estimated from the weekly averaged filters analyzed by XRF using a mass ratio of sulfate to sodium in seawater of 0.25.^70^ The mass concentration of SS-chloride was calculated as (Na × (MW_Cl_/MW_Na_) × CE_ss_), where CE_ss_ is the HR-ToF-AMS sea salt collection efficiency with a value of about 0.02.^71^ The low concentrations of NSS-chloride (0.036–0.055 μg m^–3^ or <2% of PM_1_) corresponded to uncertainties in NSS-chloride mass concentrations of 27% based on the detection limit relative to the mean, which were considered not sufficiently accurate for analysis at time scales of a day or less and are only included for monthly averages.

### 2.4 Back-Trajectory and Land–Sea Breeze Analysis

In this study, 48 h ensemble-mean back-trajectories from the ARMTRAJ value-added product were used for both Scripps Pier and Mt. Soledad.^72^ ARMTRAJ uses ensemble runs and multilayer coverage with high-resolution ERA5 reanalysis to provide robust back-trajectories for ground-based measurements. At Scripps Pier, surface air mass trajectories (ARMTRAJSFC) were retrieved for every 3 h originating at 0–50 m above mean sea level using the Hybrid Single-Particle Lagrangian Integrated Trajectory (HYSPLIT) model^73^ with the fifth generation European Centre for Medium-Range Weather Forecasts (ECMWF) atmospheric reanalysis (ERA5)^74^ at 0.25 degree and 1 h spatial and temporal resolutions. The trajectories were clustered using a *k*-means clustering algorithm based on their geographical coordinates (longitude and latitude) to identify mean trajectories during EPCAPE to represent the origin of air masses.^75^ At Mt. Soledad, ARMTRAJSFC were used when Mt. Soledad was within the planetary boundary layer (PBL) height estimated by the LightGBM method. The LightGBM^76^ machine learning algorithm was trained using seven years of radiosonde-derived PBLH data from the ARM Southern Great Plains (SGP) site. Free troposphere back-trajectories (ARMTRAJPBL) were used when Mt. Soledad was above the Richardson number-based PBL height (Figure S1). For each site, five different clusters of back-trajectories were identified and are termed Coastal Northwesterly (CNW), Los Angeles-Long Beach (LALB), Southerly (SOU), Easterly (EAS), and Marine Westerly (MWE). Mass concentrations of NR-organics, NR-nitrate, rBC, NSS-chloride, NSS-sulfate, NR-ammonium, sea salt, and dust averaged over 3 h were categorized by the five identified air mass origins (Figure S4) based on the start time of back-trajectories.

Land and sea breezes were evaluated based on three metrics, namely wind direction, wind speed, and time of day. Only times with wind speeds exceeding 0.5 m/s were considered because lower wind speeds were too variable to retrieve a consistent direction. The criteria for sea breeze were a wind direction of 190°–360° from 10 am to 6 pm local time, which occurred for 2042 h (26% of EPCAPE, excluding rain and clouds at Mt. Soledad), while the land breeze was defined as a wind direction of 0°–190° from 8 pm to 8 am local time, which occurred for 1658 h (21% of EPCAPE, excluding rain and clouds at Mt. Soledad).

## 3 Results and Discussion

The average PM_1_ mass concentration at Scripps Pier during EPCAPE was 3.79 ± 3.16 μg m^–3^, with NR-organics being the most abundant aerosol component, accounting for 48% of the PM_1_ concentration (1.83 ± 1.92 μg m^–3^). This PM_1_ component was followed by NSS-sulfate at 20% (0.75 ± 0.67 μg m^–3^), NR-ammonium at 11% (0.42 ± 0.43 μg m^–3^), and NR-nitrate at 8% (0.31 ± 0.58 μg m^–3^). At Mt. Soledad, the average PM_1_ mass concentration was similar to that at Scripps Pier, at 3.80 ± 3.00 μg m^–3^ with the highest contribution from NR-organics at 51% (1.96 ± 2.10 μg m^–3^), followed by NSS-sulfate at 21% (0.80 ± 0.69 μg m^–3^), NR-ammonium at 8% (0.32 ± 0.27 μg m^–3^), and NR-nitrate at 8% (0.30 ± 0.40 μg m^–3^). At both sites, NSS-chloride, rBC, sea salt, and dust were minor components at 1–3% of PM_1_. These mass concentrations are listed in Table 1 and are defined to be the “EPCAPE averages” for Mt. Soledad and Scripps Pier from 15 February 2023 to 14 February 2024, including all available measurements and excluding times with rain and clouds at Mt. Soledad and number concentrations exceeding 8000 cm^–3^.

**Table 1:**
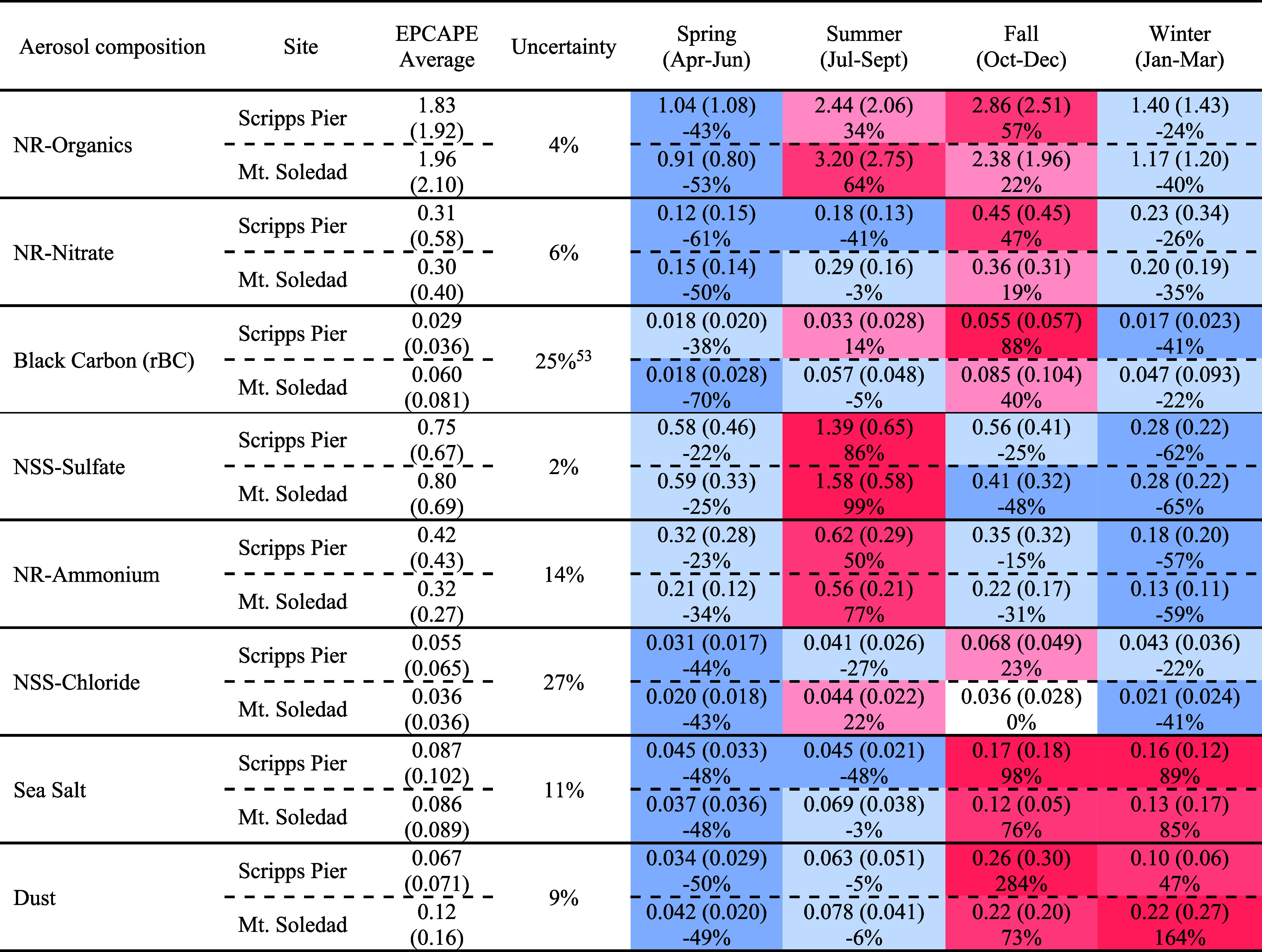
Average Mass Concentrations (μg m^–3^) of NR-Organics, NR-Nitrate, Black Carbon, NSS-Sulfate, NR-Ammonium, NSS-Chloride, Sea Salt, and Dust during EPCAPE (EPCAPE Average) and Separately for Each Season, with Standard Deviations Given in Parentheses, Excluding Times with Rain and Clouds at Mt. Soledad and Number Concentrations Exceeding 8000 cm^–3^ ^a^

a Seasonal differences are statistically significant (*p* < 0.05, ANOVA) for all 8 components. The percentage difference of the seasonal average concentrations from the EPCAPE average for each site is included below the mass concentrations, with blue shading indicating negative differences and red indicating positive differences. The percent uncertainty was calculated based on the detection limit of each composition to EPCAPE average mass concentrations.

### 3.1 Similar Source-Related Monthly Changes in Aerosol Composition at Scripps Pier and Mt. Soledad

The monthly chemical mass concentrations at Scripps Pier and Mt. Soledad show similar seasonal patterns and magnitudes for the two sites (Figure 1 and Table 1). The spring mass concentrations averaged from April to June are lower than the EPCAPE averages, with an NR-organics mass concentration of 1.04 μg m^–3^ at Scripps Pier and 0.91 μg m^–3^ at Mt. Soledad. In summer (July to September), NSS-sulfate mass concentrations at the two sites were higher than the EPCAPE averages at 1.39 and 1.58 μg m^–3^, and NR-ammonium mass concentrations were also higher at 0.62 and 0.56 μg m^–3^, for Scripps Pier and Mt. Soledad, respectively. In fall (October to December), the mass concentrations of NR-nitrate, NR-organics, and rBC were 0.36–0.45, 2.38–2.86, and 0.055–0.085 μg m^–3^, respectively, with these concentrations being higher than the EPCAPE averages by 19 to 88%.

**Figure 1:**
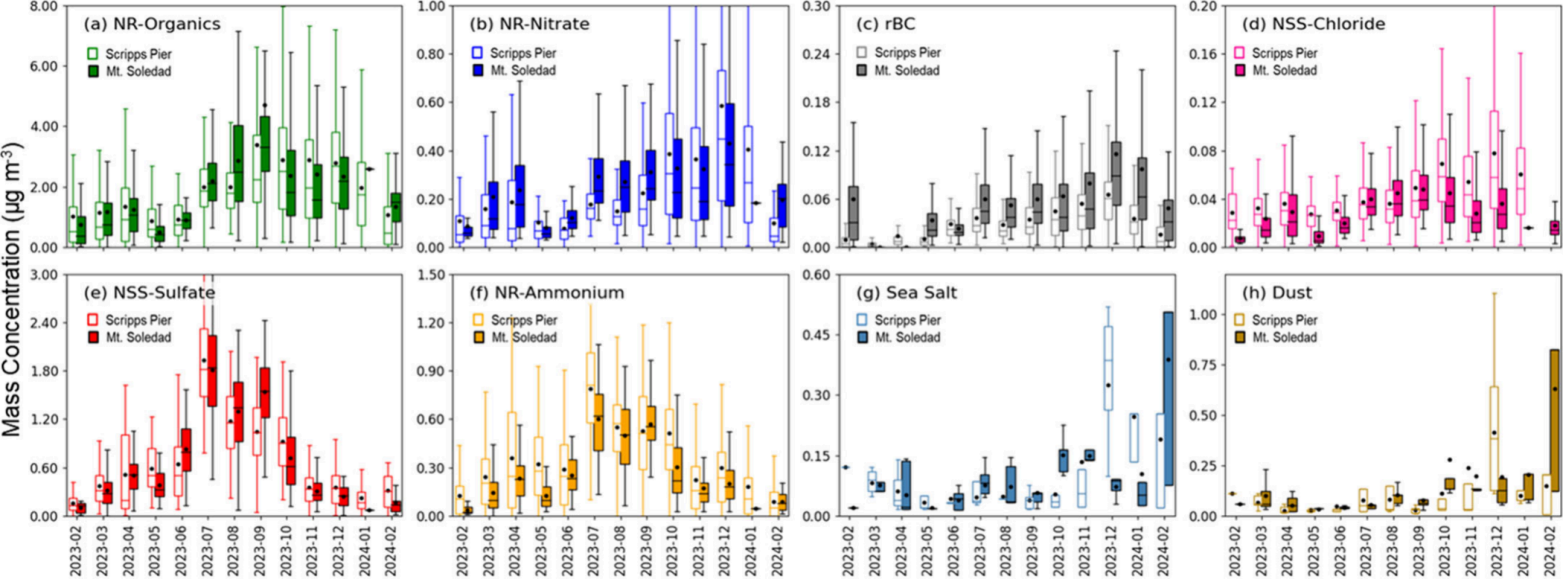
Monthly average mass concentrations (μg m^–3^) of (a) NR-organics, (b) NR-nitrate, (c) rBC, (d) NSS-chloride (e) NSS-sulfate, (f) NR-ammonium, (g) sea salt, and (h) dust in PM_1_ at Scripps Pier (empty) and Mt. Soledad (filled), excluding times with rain and clouds at Mt. Soledad and number concentrations exceeding 8000 cm^–3^. The averages are calculated from 3 h averages at each site, with black dots representing the monthly means. The boxes indicate the interquartile range (IQR), with the middle line marking the median, the top edge representing the 75th percentile, and the bottom edge representing the 25th percentile. The whiskers extend to the maximum and minimum non-outlier values.

There are small contributions from sea salt and dust to the PM_1_ composition. Sea salt (Figure 1g) and dust (Figure 1h) mass concentrations were consistently low until September and then higher from November through February, reaching maximum monthly concentrations of 0.3 (±0.1) μg m^–3^ for sea salt and 0.4 (±0.3) μg m^–3^ for dust in December. The site-to-site differences for sea salt and dust concentrations are not statistically significant because of the small number of daily samples that was analyzed.

PM_1_ mass concentration from summing the measured components was 4.8 (±1.9) μg m^–3^ at Scripps Pier and 5.9 (±2.1) μg m^–3^ at Mt. Soledad in summer (July to September). These NR mass concentrations are slightly lower than those reported between August and October in 2006 (7 μg m^–3^)^20^ and in 2009 (8.4 μg m^–3^)^18^ at Scripps Pier. Likely, the lower concentrations during EPCAPE were because major wildfire events on 28 September 2006 and 29 August 2009 contributed to higher concentrations in those years. In summer 2008, the concentration for a nonfire period was 2.0 ± 0.72 μg m^–3^ for NR-organics, which is comparable to the NR-organics concentration (2.07 ± 0.12 μg m^–3^) for August 2023 during EPCAPE.

Previous studies^18–23^ have highlighted regional influences of anthropogenic aerosols in southern California, with transport from nearby upwind sources such as the Los Angeles-Long Beach (LALB) area, Riverside, and Tijuana impacting aerosol compositions in La Jolla and San Diego. The seasonal differences in aerosol composition can largely be explained by the changes in the frequency of different back-trajectories (Figure 2). Back-trajectories for Mt. Soledad and Scripps Pier came from the same upwind region for 91% of the time, when both sites were within the boundary layer (Figures S1 and S2). During April–June, back-trajectories were CNW for 86–93% of the time and were associated with concentrations of NR-organics (1.04 ± 1.08 and 0.91 ± 0.80 μg m^–3^), NR-nitrate (0.12 ± 0.15 and 0.15 ± 0.14 μg m^–3^), and rBC (0.018 ± 0.020 μg m^–3^), which were 38–70% lower than the EPCAPE averages. The mass concentrations of NSS-sulfate (Figure 1e) and NR-ammonium (Figure 1f) were 86–99% higher than the EPCAPE averages from July to September at both Scripps Pier and Mt. Soledad because CNW back-trajectories occurred more than 71% of the time and brought high sulfate precursor contributions from ocean phytoplankton sources during warm summer months.^77^ LALB back-trajectories occurred 18–19% of the time and had NR-organics and NR-nitrate concentrations that were 50–134% higher than the EPCAPE averages (Table S2). During the fall, EAS back-trajectories brought rBC mass concentrations that were 65% higher than the EPCAPE averages at 0.048–0.100 μg m^–3^ because of contributions from inland urban sources. SOU back-trajectories were slightly lower than EPCAPE averages, except for NR-nitrate and rBC, which were 3–19% higher. EAS back-trajectories were associated with higher average concentrations of 0.28–0.65 μg m^–3^ dust and 0.26–0.30 μg m^–3^ sea salt in late fall and early winter (Table S2). The fall and winter mix of back-trajectories from all directions resulted in higher average concentrations of dust and sea salt that were largely driven by LALB and EAS back-trajectories.

**Figure 2:**
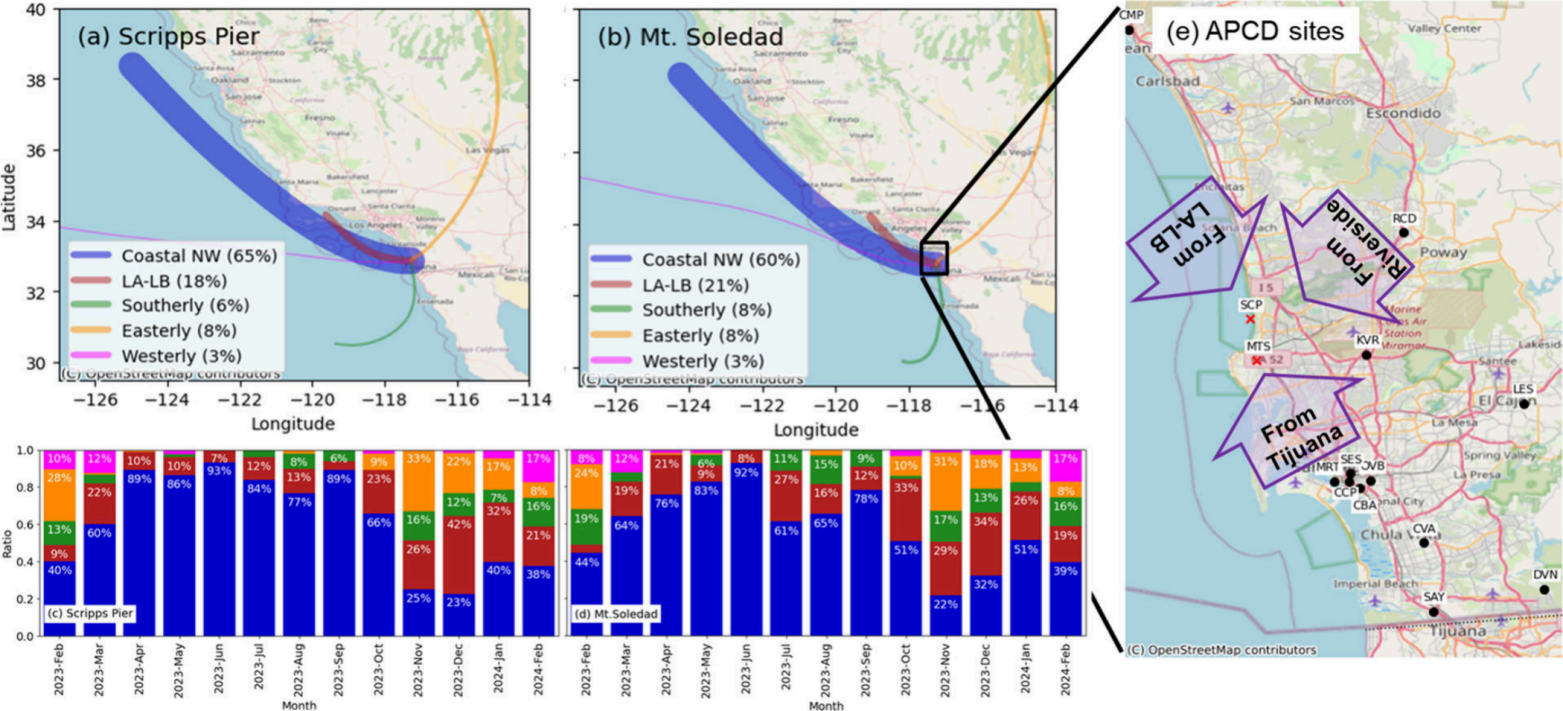
Five cluster centroids of back-trajectories identified for EPCAPE at (a) Scripps Pier (SCP) and (b) Mt. Soledad (MTS): Coastal NW clusters (CNW, blue), Los Angeles-Long Beach (LALB, red), Southerly (SOU, green), Easterly (EAS, orange), and Marine Westerly (MWE, magenta). The thickness of the clustered back-trajectories in (a) and (b) represents the relative contribution of each cluster during EPCAPE, for which the fraction of each cluster is shown by month at (c) Scripps Pier and (d) Mt. Soledad. Scripps Pier uses ARMTRAJSFC, and Mt. Soledad uses ARMTRAJSFC when the planetary boundary layer height is above the site and ARMTRAJPBL otherwise. The black box in (b) corresponds to the boundaries of (e) showing the locations of Air Pollution Control District (APCD) sites in San Diego, and the purple arrows indicate nearby upwind regions.

The similarity of seasonal patterns and magnitudes of chemical mass concentrations at Scripps Pier and Mt. Soledad (Figure 1 and Figure S1) shows that emissions from upwind regions likely had a large contribution to the monthly average aerosol composition that overwhelmed any local differences between the sites. rBC and NR-nitrate are likely mostly driven by vehicle emissions, which are highest in upwind urban regions,^78–80^ such as the LALB urban port megacity to the north as well as the cities of Riverside and San Diego to the northeast and south. Submicron NSS-sulfate increased in the summer during CNW back-trajectories, likely associated with biogenic sulfate from phytoplankton,^77^ with concomitant ammonium concentrations.^9^ Submicron sea salt had low concentrations (<0.15 μg m^–3^) for most of the year, consistent with the low wind speeds offshore during CNW back-trajectories, but interestingly, the sea salt concentration was also higher for EAS back-trajectories that passed near the Salton Sea (Figure S4 and Table S2). The similarities and differences for other back-trajectory regions are discussed in Text S1, which also describes the regional context provided by comparison to inland Air Pollution Control District (APCD) sites (Figures S5 and S6).

Organic composition can be a good indicator of individual and mixed aerosol sources.^81–83^ Comparing the 3 h average mass spectra of NR-organics measured by HR-ToF-AMS at Mt. Soledad and ACSM at Scripps Pier shows strong similarities among the organic sources for each back-trajectory category at both sites (Figure 3). Cosine similarity is calculated as the dot product of two vectors and is used to show the degree to which the mass spectra are similar for each site, between sites, and between different back-trajectory clusters. The median cosine similarity of CNW, LALB, SOU, and EAS back-trajectories was above 0.88 for both sites, illustrating the overall similarities in NR-organics *m*/*z* spectra from each back-trajectory region (Table S1). Between sites, the means of the cosine similarities varied between 0.88 and 0.98 depending on the back-trajectories, suggesting that the sources of NR-organics were quite similar for both sites for each back-trajectory cluster. These 12 month average values are comparable to the cosine similarities of >0.9 that were considered similar by past studies that compared individual measured spectra to positive matrix factorization (PMF) factors.^81,83^

**Figure 3:**
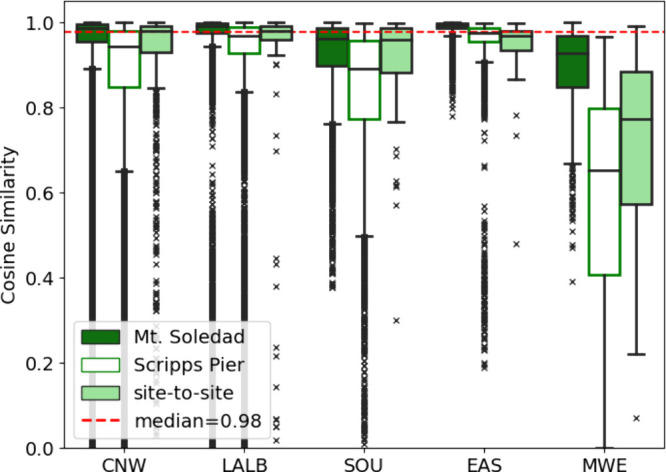
Box plots of cosine similarities of 3 h average NR-organics mass spectra within the categorized back-trajectories at Mt. Soledad (filled green) and Scripps Pier (empty), excluding times with rain and clouds at Mt. Soledad and number concentrations exceeding 8000 cm^–3^. The solid light green boxes show site-to-site comparisons. The red dashed line indicates the median value of the site-to-site comparison. Boxes show the interquartile range (25th to 75th percentile). The middle line marks the median, and the whiskers extend to the maximum and minimum non-outlier values. The cosine similarity is calculated from unit mass resolution (UMR) signals for *m*/*z* 40–110 within and between the two sites for each back-trajectory cluster.

### 3.2 Contributions of Semivolatile Inorganic Components

Consistent with the monthly concentrations at the two sites being largely similar because of the similar sources from each back-trajectory category, 3 h average mass concentrations of NR-organics and NSS-sulfate at Mt. Soledad were strongly correlated with those measured at Scripps Pier (*r* = 0.82 for NR-organics and *r* = 0.73 for NSS-sulfate, Figure 4). In contrast, NR-nitrate (*r* = 0.63), NR-ammonium (*r* = 0.54), and rBC (*r* = 0.62) had only moderate correlations between the sites. These 3 h differences could be caused by the time lag between the two sites, but a lagged correlation analysis shows that this effect is an hour or less for both land and sea breeze conditions and does not substantially improve the correlation (Table S6). Another potential cause of these shorter time scale differences is local emissions between the two sites, and these likely explain the rBC differences, as discussed in section 3.3.

**Figure 4:**
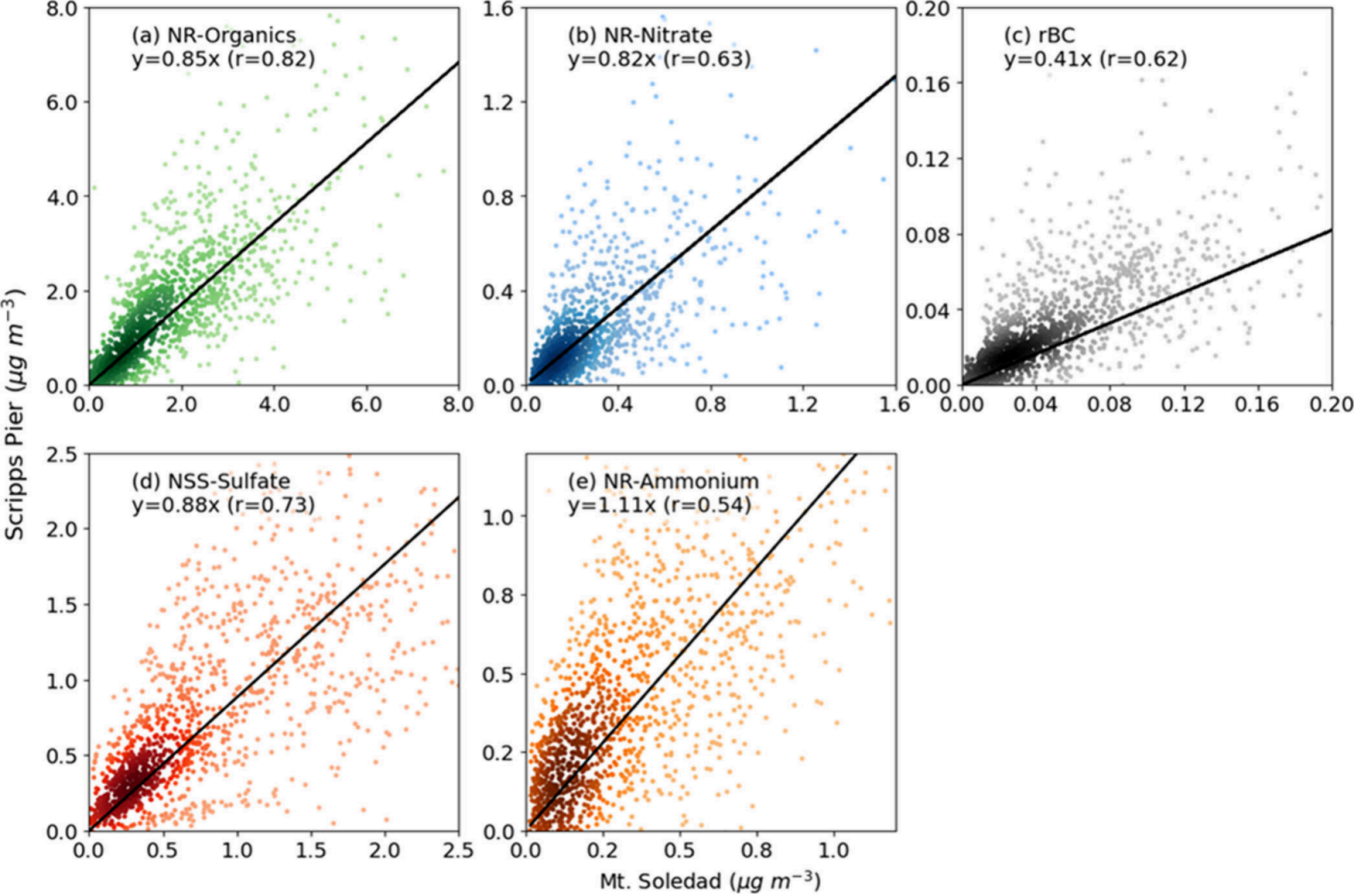
Scatter plots and least-squares regression lines of time series for 3 h average mass concentrations of (a) NR-organics, (b) NR-nitrate, (c) rBC, (d) NSS-sulfate, and (e) NR-ammonium at Mt. Soledad compared to those at Scripps Pier for all back-trajectories, excluding times with rain and clouds at Mt. Soledad and number concentrations exceeding 8000 cm^–3^. Pearson correlations were significant (*p* < 0.05) for all five components.

The lower correlation coefficients for NR-nitrate (*r* = 0.63) and NR-ammonium (*r* = 0.54) mass concentrations compared to those for NR-organics and NSS-sulfate mass concentration suggests that the lower correlations are driven by the semivolatile properties of ammonium nitrate, which partitions to particles from the gas phase for lower temperature and higher relative humidity conditions.^24,26^ This interpretation is supported by the moderate positive correlation of the daily average site-to-site differences in NR-nitrate (*r* = 0.45) concentrations to the site-to-site difference in RH (%) and the moderate negative correlation (*r* = −0.50) to the site-to-site difference in temperature (°C), when higher concentrations at Scripps Pier than Mt. Soledad are excluded to remove times with large emissions at Scripps Pier (Figure 5). The moderate correlation (*r* = 0.54) and lower mass concentrations at Mt. Soledad of NR-ammonium are likely the result of the combination of semivolatile contributions with losses to vegetative and other land surfaces.^84,85^ Daily average site-to-site differences are used in this comparison to minimize contributions from the time lag between the sites and from night–day differences associated with land–sea breezes. This evaluation excludes times when the difference in daily average mass concentration between Mt. Soledad and Scripps Pier was negative because those times are interpreted as having large emissions at Scripps Pier. Times when the absolute difference in site-to-site concentrations is lower than the detection limit for the component (Table 1) are excluded because the relative error from subtracting to evaluate the difference is large. Excluding times with small and negative site-to-site differences necessarily excludes times when the effect of the temperature difference (combined with local emissions between the sites) outweighs the effect of the relative humidity difference, limiting this analysis to only those times when the relative humidity difference is smaller. Nonetheless, the comparison provides evidence of the micrometeorological modulation of site-to-site differences in nitrate concentration.

**Figure 5:**
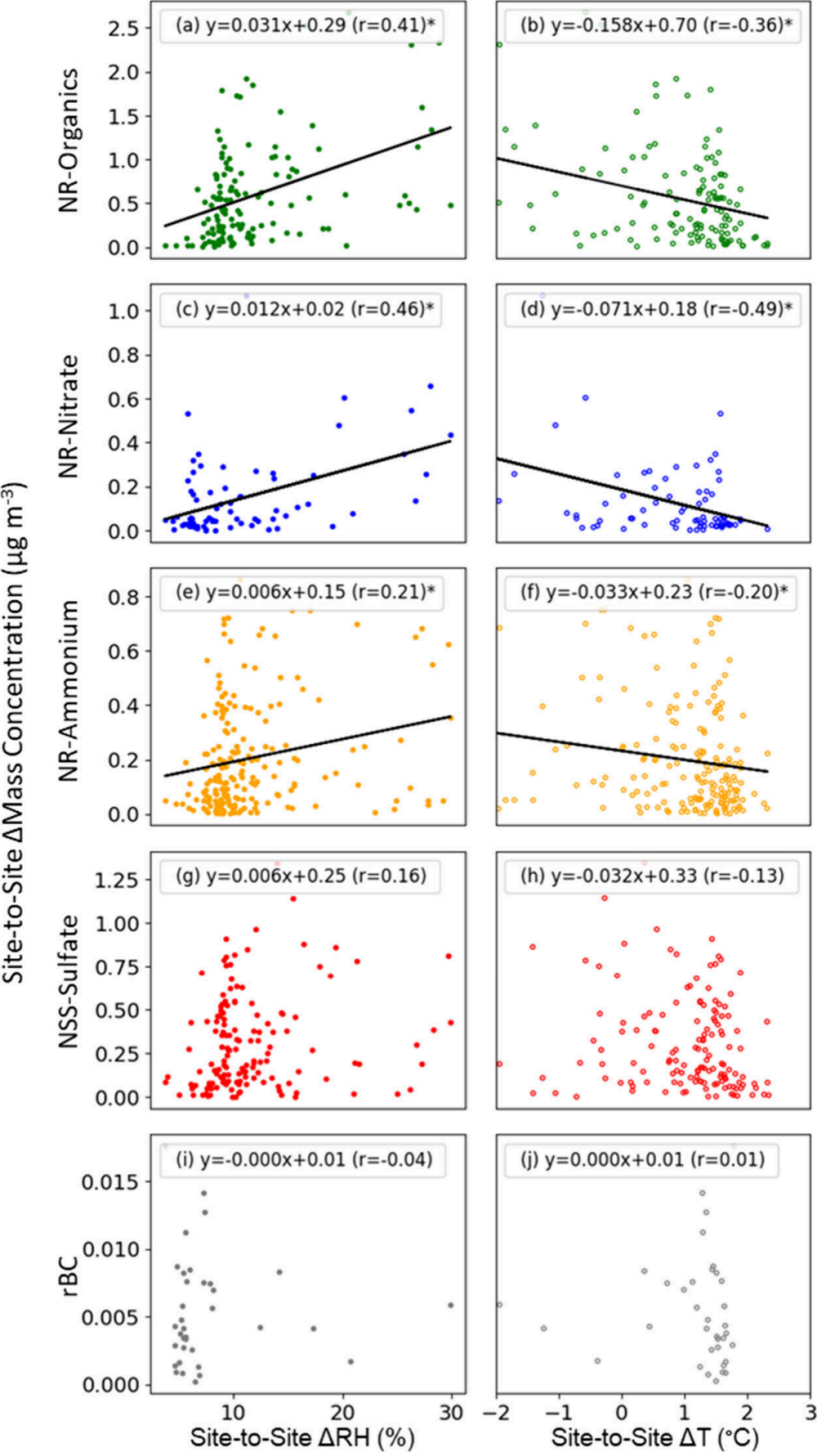
Scatter plots of the daily average mass site-to-site difference for NR-organics, NR-nitrate, NR-ammonium, NSS-sulfate, and rBC mass concentrations to the site-to-site difference of relative humidity (RH) and temperature between the two sites for all back-trajectories, excluding times with rain and clouds at Mt. Soledad and number concentrations exceeding 8000 cm^–3^. Site-to-site differences are calculated by subtracting Mt. Soledad values from Scripps Pier values. Asterisks (*) indicate statistically significant correlations (*p* < 0.05). NSS-chloride is not included because of its large uncertainty. NR components had 275 days with at least 3 h of measurements at both sites; of those, the number of days that had a mass difference higher than the detection limit was 111 for organics, 68 for nitrate, 178 for ammonium, and 136 for sulfate. rBC had 265 days available, and only 10 days are left after filtering the days when the difference is less than the error.

This dependence of site-to-site difference in NR-nitrate concentrations represents changes of +0.01 μg m^–3^ per percentage RH and −0.07 μg m^–3^ per degree Celsius. The change in the site-to-site difference in ammonium concentration is similar (after accounting for the lower mass per mole of ammonium per mole of nitrate) at +0.01 μg m^–3^ per percentage site-to-site difference in RH and −0.03 μg m^–3^ per degree Celsius site-to-site difference in temperature. However, the correlations for the daily average site-to-site differences in NR-ammonium to the site-to-site relative humidity difference and site-to-site temperature difference are weaker (*r* = 0.20 and *r* = −0.21, respectively) than for the site-to-site difference in NR-nitrate, likely because some ammonium is associated with the less volatile sulfate (Table S3). Site-to-site differences in NR-organics show both a strong correlation (*r* = 0.82) between sites and moderate correlations to site-to-site difference in temperature and relative humidity (*r* = −0.30 and *r* = 0.36), suggesting that it consists largely of nonvolatile contributions but includes some less volatile contributions. As expected, there is no relative humidity or temperature site-to-site difference for rBC or NSS-sulfate.^24^

### 3.3 Effects of Land–Sea Breezes and Local Traffic on Aerosol Composition

Consistent with many coastal urban locations,^31,32,34^ the land–sea breeze pattern corresponded to changes in the wind direction at Scripps Pier and Mt. Soledad (Figure S7), bringing contrasting aerosols from offshore during the day and from inland at night. This change in wind direction means that emissions from inland sources, such as traffic and urban emissions, are transported to the EPCAPE sites at night, while emissions from offshore sources are transported to the sites during the day. In addition, there is a difference between the two sites from “local” sources, quantified here as the sources that contribute emissions between Scripps Pier (330 m offshore) and Mt. Soledad (3 km inland). This difference between the two sites is likely caused by the local traffic emissions between and surrounding the two sites.

EPCAPE meteorological measurements showed a frequent pattern of daytime sea breezes from the northwest and nighttime land breezes from the east, with the transitions in wind direction occurring in the mornings between 8 and 10 am and in the evenings between 6 and 8 pm (local time, Figure S7). Wind speed was generally higher during the sea breeze, consistent with the prevailing larger-scale CNW flow. The land breeze criteria were satisfied for 1658 h during EPCAPE and the sea breeze criteria for 2042 h, yielding significant differences in mean concentration for rBC of 0.012–0.017 μg m^–3^ and for NR-nitrate of 0.07–0.08 μg m^–3^. The mean rBC concentration was 59% higher at Scripps Pier and only 20% higher at Mt. Soledad for land breezes compared to sea breezes (Figure 6), reflecting the cleaner sea breeze concentrations at Scripps Pier since it is offshore. Wind speeds were also stronger and more consistent at Scripps Pier (Figure S7), supporting the greater land–sea difference relative to Mt. Soledad for most components. For only CNW back-trajectories, the land–sea breeze differences are lower because the gradient at the coast is weakened by the upwind air, bringing offshore air further onshore during the day that dilutes the emissions brought by the land breeze before they reach Scripps Pier. The land–sea breeze differences in NR-nitrate were lower than for rBC because the gradient in concentration is dampened by the time needed to form NR-nitrate, with only a 24% difference at Scripps Pier and 30% difference at Mt. Soledad. The NSS-sulfate concentration was 3–15% lower for land breeze compared to sea breeze at both sites, consistent with its ocean-related sources^77^ (Table S5). The daily transitions in the land–sea breeze effect, which nearly coincided with expected morning and evening traffic patterns, also meant that diurnal patterns in aerosol composition were weak at both sites (Figure S9).

**Figure 6:**
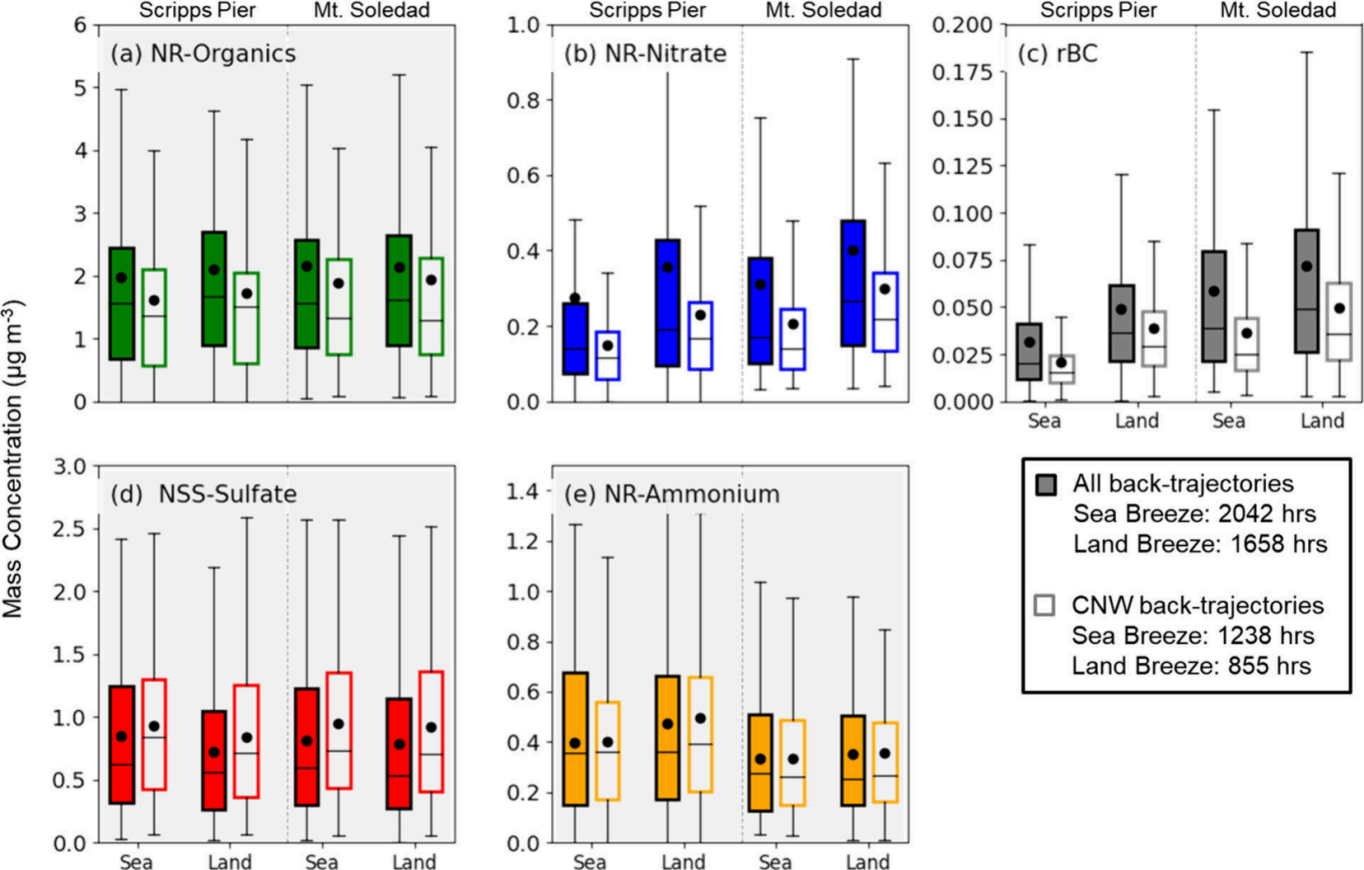
Comparisons of average mass concentrations of (a) NR-organics, (b) NR-nitrate, (c) rBC, (d) NSS-sulfate, and (e) NR-ammonium for all back-trajectories (filled, 2042 h for sea breeze and 1658 h for land breeze) and CNW only (empty, 1238 h for sea breeze and 855 h for land breeze) at Mt. Soledad and Scripps Pier, calculated from 30 min averages and excluding times with rain and clouds at Mt. Soledad and number concentrations exceeding 8000 cm^–3^. Background shading indicates compounds for which the land–sea breeze effects were not significant, based on a *t* test for both Scripps Pier and Mt. Soledad. The percentage differences for land–sea breeze effects are listed in Table S5. The symbols represent mean values, while the boxes show the median, with the upper (75th percentile) and lower (25th percentile) quartiles, with caps marking the maximum and minimum non-outlier values.

For all back-trajectories during EPCAPE, the night–day difference was significant only for rBC at Scripps Pier with a 15% difference (Table 2). The rBC night–day difference is likely significant because rBC is directly emitted from residential and traffic sources and had a sharp gradient in concentration at the coast,^47,86,87^ while other inland components like NR-nitrate that are formed by secondary reactions have less distinct gradients in concentration because they form only after reactions that require additional time in the atmosphere, with that additional time allowing for more mixing. During EPCAPE, 262 days had >6 h sea breeze following nights with >6 h land breeze, and these days had a 16% night–day difference in rBC at Scripps Pier compared to the remaining 61 days that had only an 8% difference. This difference indicates that the land–sea breeze was the main cause of the night–day difference for rBC, with the land breeze bringing more inland sources directly emitted from residential and traffic sources. In contrast, NSS-sulfate was 2–14% lower at night (Table 2) because of sea breezes bringing higher ocean sources during the day. NR-organics and NR-ammonium mass concentrations during EPCAPE showed night–day differences <10%, indicating that photochemical reactions, traffic or other activity patterns, and land–sea breezes had small and variable effects on their concentrations. The photooxidation that would normally increase daytime aerosol concentrations occurred when sea breezes brought cleaner air with fewer pollutants to oxidize. Enhanced emissions from traffic are expected in the morning and early evening, but these times overlapped with the transitions from the sea to land breezes, resulting in no clear diurnal patterns (Figure S9). In addition, the frequent cloudiness^77^ meant that there was no photochemically driven diurnal cycle in NR-nitrate or NR-organics mass concentrations, as is often observed in urban areas.^88,89^

**Table 2:**
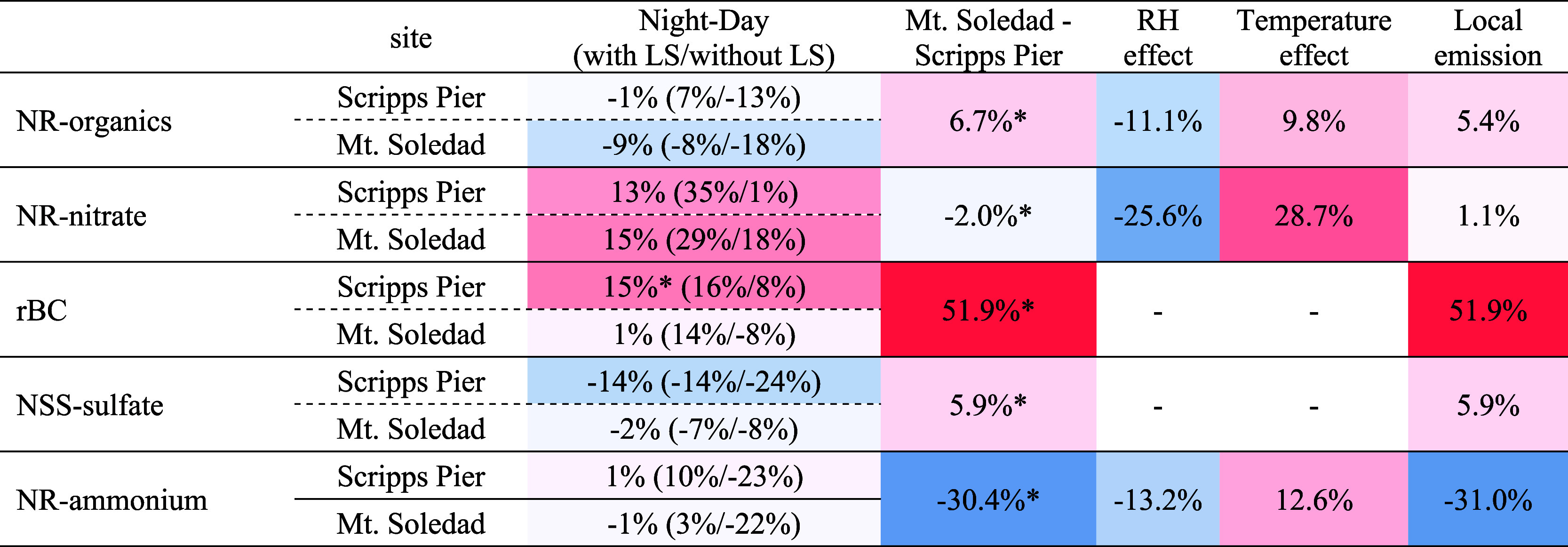
Percentage Differences of NR-Organics, NR-Nitrate, rBC, NSS-Sulfate, and NR-Ammonium for Night–Day (Night–Day Difference), Mt. Soledad–Scripps Pier (Site-to-Site Difference), RH Effects, Temperature Effects, and Local Emissions for All Back-Trajectories^a^

a Measurements are averaged for 3 h, and percentage differences are calculated as the difference at each site, excluding times with rain and clouds at Mt. Soledad and number concentrations exceeding 8000 cm^–3^ relative to EPCAPE average concentrations at Mt. Soledad (given in Table 1). Asterisks (*) denote significant differences based on *t* test (*p*-value < 0.05). Night–day differences are calculated for 6 am to 6 pm local, with values shown for days with and without consecutive land–sea breezes (in parentheses). Local emissions are calculated as the difference between Mt. Soledad and Scripps Pier, adjusted for RH and *T* effects. Average *T* and RH values at Scripps Pier (16.3 °C, 80%) and Mt. Soledad (15.1 °C, 73%) are applied to the slopes of fitted lines in Figure 5 that are significant, namely for NR-organics, NR-nitrate, and NR-ammonium mass concentrations.

The contributions of local emissions from activities between Scripps Pier and Mt. Soledad are most evident in the primary rBC concentration differences (Table 2). The EPCAPE average rBC was 0.060 ± 0.081 μg m^–3^ at Mt. Soledad, almost twice as high as the EPCAPE average at Scripps Pier of 0.029 ± 0.035 μg m^–3^, consistent with the low slope of 0.41 and moderate correlation of *r* = 0.62 (Figure 4). This difference is attributed to the local traffic-related emissions since Mt. Soledad is within 10 m of roads, while Scripps Pier is 330 m offshore. Further evidence of the impact of local traffic-related emissions is provided by six tracers of vehicle-related emissions (Fe, Ti, Mn, Cu, Zn, and Pb), which were significantly higher (*p* < 0.05) at Mt. Soledad compared to Scripps Pier (Figure 7 and Figure S10). Fe, Ti, and Mn are weakly but negatively correlated with wind speed, indicating that they are concentrated by low winds because they are emitted primarily by nearby sources so that they are highest when winds are lowest. Fe has been reported as a tracer of both tire wear and brake wear,^46^ and Ti and Mn have been reported as brake wear tracers.^90–92^ These brake wear tracers are produced at high brake temperatures, and continuous braking can increase fine particle fragmentation, although the highest concentrations are observed in coarse particles.^40,93^ The road adjacent to the Mt. Soledad site, Via Capri, has high traffic counts (Figure S12) and an average grade of 9.4% (446 ft/0.9 mi) that increases in steepness to 12–13% around the site,^94^ leading to frequent braking by motorists. Zn and Cu are also brake wear tracers due to their high fraction in brake linings^46,91,95–97^ and also had significantly higher concentrations at Mt. Soledad (Figure S10), but their correlation with Pb (Figure S11) and lack of negative correlation with wind speed suggest their sources may come largely from fuel combustion, which is emitted from the larger surrounding area rather than concentrated at the steep nearby roads. It is possible that recent California regulations to reduce Cu in runoff from urban areas by reducing the Cu composition in brake pads^98^ have made the Cu contributions to brake wear smaller in some vehicles.

**Figure 7:**
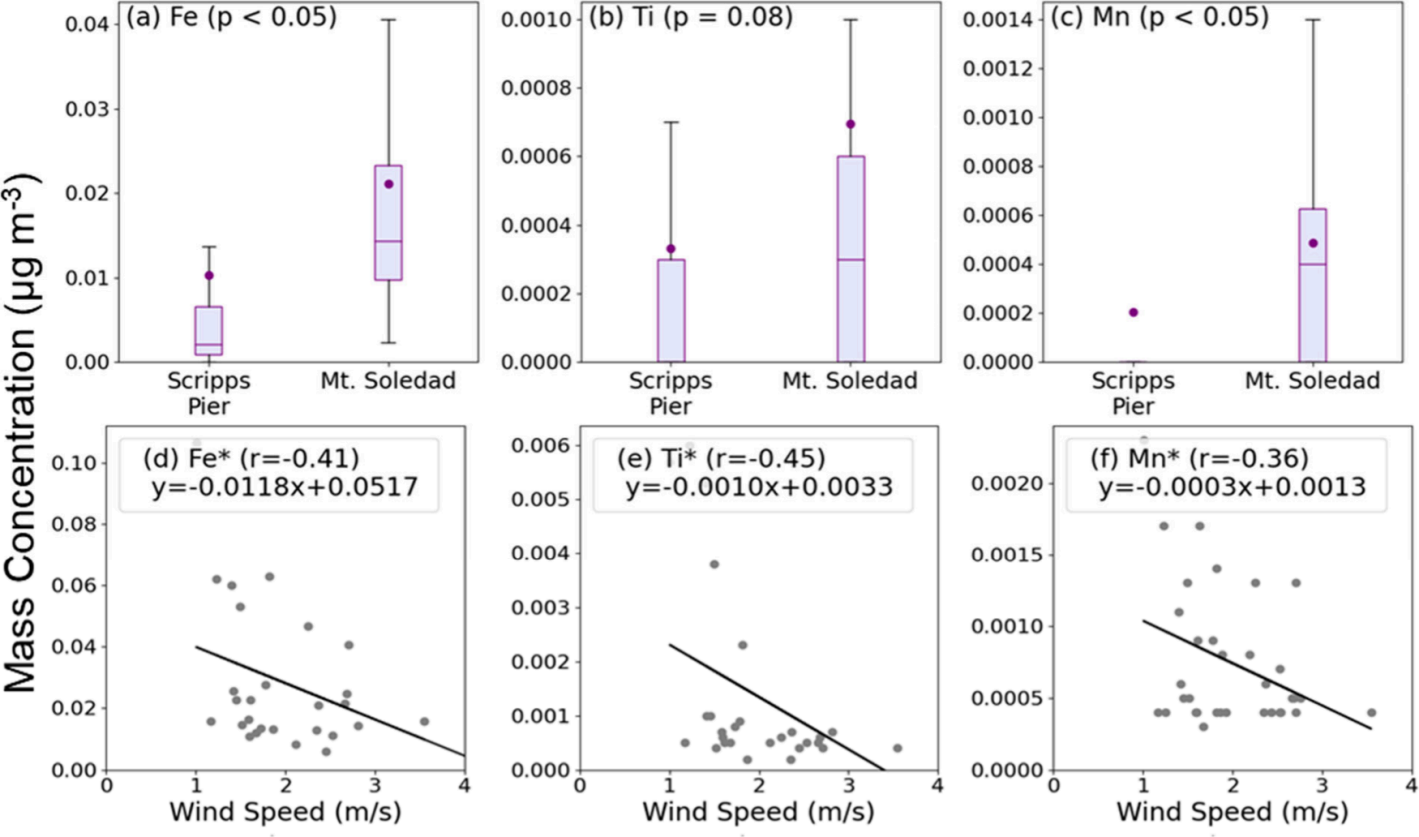
Average and range of mass concentrations for (a) Fe, (b) Ti, and (c) Mn at Scripps Pier and Mt. Soledad, from three daily filters each week, excluding days with rain and clouds at Mt. Soledad and number concentrations exceeding 8000 cm^–3^. The dot symbols represent mean values; boxes show the interquartile range (25th to 75th percentile), with the line indicating the median. Whiskers extend to the maximum and minimum non-outlier values. *p*-values from *t* tests indicate the significance of the differences between the two sites. Dependence of (d) Fe, (e) Ti, and (f) Mn mass concentrations on wind speed (m/s) at Mt. Soledad, with fitted lines and Pearson’s correlation coefficients (*r*) shown. Asterisks (*) indicate statistically significant correlations (*p* < 0.05).

## 4 Conclusions

EPCAPE aerosol mass concentrations illustrate that PM_1_ aerosol concentrations in coastal regions are largely determined by the sources in upwind regions, which are controlled by the large-scale meteorology that controls the back-trajectories. The occurrence of different back-trajectory categories was similar at Mt. Soledad and Scripps Pier for 91% of the time during EPCAPE, resulting in very similar mass concentrations at both sites for NR components on monthly time scales. CNW back-trajectories were the most frequent, occurring more than 71% of the time from April to June, with mass concentrations of NR-organics, NR-nitrate, and rBC that were 38–70% lower than the EPCAPE averages for both sites because the offshore trajectories did not pass over nearby upwind urban areas. In contrast, NSS-sulfate was 14–18% higher than the EPCAPE average for CNW back-trajectories at each site, reflecting the larger contributions from ocean rather than land sources. The LALB, SOU, and EAS back-trajectories brought air masses from nearby urban regions, with LALB trajectories bringing mass concentrations that were higher than the EPCAPE average by 50% or more for NR-organics, NR-nitrate, and rBC concentrations because of the high density of transportation sources in the Los Angeles and Long Beach urban port areas. Regional APCD monitoring sites across San Diego County also show differences by back-trajectories with elevated levels of rBC, NO, CO, and O_3_ linked primarily to LALB, SOU, and EAS back-trajectories. These significant differences in aerosol mass concentration with back-trajectories indicate that the upwind urban sources control the monthly aerosol composition at both sites and regionally. In addition, the mean cosine similarity of the *m*/*z* spectra of NR-organics between the two sites was 0.98, showing very similar organic chemical composition and sources for most of the time.

On the shorter time scales of 30 min, 3 h, and 1 day, Mt. Soledad concentrations correlated moderately with those measured at Scripps Pier for semivolatile NR-nitrate (*r* = 0.63) and NR-ammonium (*r* = 0.54) and strongly for less volatile NR-organics and NSS-sulfate (*r* = 0.73–0.82). These site-to-site differences in NR-nitrate concentrations are explained in part by the weak to moderate correlations of NR-nitrate mass concentrations to site-to-site differences in temperature and relative humidity, showing evidence that local meteorological differences shift the partitioning of semivolatile components. The implication of this result is that the partitioning of nitrate to particles changed by a site-to-site difference of +0.01 μg m^–3^ per percentage site-to-site difference in relative humidity and by −0.07 μg m^–3^ per degree Celsius site-to-site difference in temperature at Scripps Pier compared to Mt. Soledad (with comparable changes for site-to-site difference in ammonium of 0.006 μg m^–3^ per percentage relative humidity and −0.03 μg m^–3^ per degree Celsius). These two effects partially offset each other on average since the higher relative humidity at Scripps Pier increased nitrate mass concentrations by 26%, but its higher temperature decreased nitrate mass concentrations by 29%. These dependences of site-to-site difference in particle nitrate mass concentrations on the site-to-site difference in relative humidity and temperature should be compared with those predicted in model simulations to evaluate the accuracy of available aerosol parametrizations.

Local sources and winds have component-specific contributions to site-to-site differences. Land breezes were identified for 1658 h and sea breezes for 2042 h of EPCAPE, during which rBC was 59% higher and NR-nitrate was 24% higher for land breezes compared to sea breezes at Scripps Pier, consistent with higher inland sources of these components. These land–sea breeze differences contributed to slightly higher average nighttime concentrations of NR-nitrate and rBC, but the night–day difference was significant only for rBC at Scripps Pier. Concentrations of rBC and tracers for brake wear were significantly higher at Mt. Soledad than at Scripps Pier, with rBC being 52% higher, consistent with the nearby local traffic emissions contributing to combustion emissions and the steepness of the roads surrounding Mt. Soledad contributing to brake wear emissions.

Overall, EPCAPE measurements show the role of air mass back-trajectories in determining monthly coastal aerosol composition across sites because the proximate upwind aerosol source regions control the region-wide aerosol chemical composition. The shorter time scale (hourly and daily) differences between the two sites provide observation-based quantification of the gas-particle partitioning of semivolatile NR-nitrate, which should be used to constrain simulations of aerosol concentrations in models. Land–sea breeze effects are significant only for primary rBC, likely because the slower secondary formation of nitrate and organic particles blurs the distinct temporal patterns of concentrations. The significant increase in rBC and brake wear tracers at Mt. Soledad relative to Scripps Pier is surprising given the short distance between the sites (∼3 km) but illustrates the disproportionate contribution of nearby traffic (<10 m) to sampled aerosols. Together these results show the relative contributions of both local and long-range sources to different time scales of observed compositions, as well as the important role of local meteorological differences in determining the partitioning of semivolatile components.

## Supplementary Material



## Notes

Additional details on measurements, methods, and results, as well as supplemental figures and tables, including cosine similarities, Pearson correlation coefficients and regression slopes, APCD site locations, lagged correlation coefficients, time series of aerosol composition, PBLH data, box and scatter plots of average mass concentrations at various time scales, density plots of wind directions and speeds, diurnal patterns for all back-trajectories, and traffic volume near the sites (PDF)
