# Supplementary material for: Nearby Sites Show Similar Upwind Sources and Differing Semivolatile Concentrations in Coastal Aerosol Particles
Source: ACS EST Air. 2025 Nov 22;2(12):2824–37. doi: 10.1021/acsestair.5c00191 (PMC12706731; doi:10.1021/acsestair.5c00191)
Supplement: Supplementary file 1 [file ea5c00191_si_001.pdf]

Supplementary Information

Nearby Sites Show Similar Upwind Sources and Differing Semivolatile Concentrations in Coastal Aerosol Particles

Sanghee Han[1], Abigail S. Williams[1], Lynn M. Russell[1], Veronica Z. Berta[1], Jeramy L. Dedrick[1], Christian Pelayo[1], Nattamon Maneenoi[1], Atsushi Osawa[1], Israel Silber[2], Damao Zhang[2], Maria A. Zawadowicz[3], Arthur J. Sedlacek III[3]

[1]Scripps Institution of Oceanography, University of California, San Diego, USA.
[2]Pacific Northwest National Laboratory, Richland, WA, USA.
[3]Brookhaven National Laboratory, Upton, NY, USA.

Correspondence to: Lynn M. Russell (lmrussell@ucsd.edu)

Text

Text S1. The Dependence of Aerosol Composition on Upwind Regions

CNW back-trajectories were the most frequent from March to October at both sites, and MWE back-trajectories were the least frequent throughout the year. CNW back-trajectories generally followed the climatological winds along the coast of California, bringing relatively clean air from multiple days over the ocean with some contributions from shipping offshore and cities onshore. These frequent CNW back-trajectories had NR-organics, NR-nitrate, and rBC mass concentrations as 72-91% of the EPCAPE average, consistent with the diluted nature of these emission sources compared to the nearby urban regions (Table S2). In contrast, sulfate is 14-18% higher than the EPCAPE average for CNW back-trajectories since the ocean sources are larger than those on land. The average mass concentrations of NR-organic, NR-nitrate, and rBC were the lowest for MWE back-trajectories because of the lack of upwind urban sources, often falling below the EPCAPE average concentrations by almost an order of magnitude.

Mass concentrations of NR-organics, NR-nitrate, rBC, and NSS-chloride were the highest at both sites for LALB back-trajectories (Fig. S4 and Table S2), showing large contributions of vehicle emissions from this "mega-city" urban-port region. The largest difference in concentrations for LALB trajectories was the near doubling of NR-nitrate over the EPCAPE average, likely associated with the densely-packed ocean-going and land-based vehicular sources at the ports.[1, 2, 3, 4] The averaged concentration of NR-organics was 50-73% higher at 2.94-3.15 μg m^{-3} for LALB back-trajectories than for EPCAPE average. This frequent transport of polluted air masses from LALB to La Jolla has been reported with estimated transit times of 12 to 36 hours for organic components.[5, 6] At Scripps Pier, rBC to NR-organics (r=0.74) and rBC to NR-nitrate (r=0.57) were correlated more strongly for LALB back-trajectories than for CNW back-trajectories (r=0.60 and 0.42) or SOU back-trajectories (r=0.49 and 0.22) (Table S3), consistent with the more consistent source profiles at LALB. At Mt. Soledad, correlations of rBC to NR-organics (r=0.46) and of rBC to NR-nitrate (r=0.45) were weaker, most likely due to the local sources of rBC at Mt. Soledad (Sect.3.3). The correlations suggest that the sources of rBC, NR-nitrate, and NR-organics are related, likely because they are primarily from co-varying emissions of shipping and trucking in the LALB port. rBC emitted from sources can also mix with organic or inorganic species, either emitted directly or formed by secondary processes.[7, 8]

The concentration of rBC, NR-nitrate, and NR-organics are slightly higher for SOU back-trajectories than for the EPCAPE average mass concentrations (Fig. S4) but the difference was not significant. For SOU back-trajectories, NR-nitrate concentration was higher by up to 16%. rBC concentration increased by up to 19%, which was smaller than for LALB back-trajectories. For SOU back-trajectories at Scripps Pier, NR-organic concentrations show a strong correlation with NR-nitrate (r=0.81) and a moderate correlation with rBC (r=0.49), although rBC is weakly correlated to NR-nitrate (r=0.22) (Table S3), suggesting that rBC emissions for vehicles in Mexico have different characteristic ratios to NO_x than for the US.[9]

As the contribution of EAS back-trajectories increases in November (Fig. S4), concentrations of rBC are higher than the EPCAPE average by 65% (Table S2), as are dust (Fig. 1(h)) and sea salt (Fig. 1(g)) concentrations. The higher and more significant difference in rBC from the EAS urban regions likely reflects the difference between primary and secondary aerosol sources, with the primary rBC forming more quickly and more continuously (including overnight) than the secondary NR-organics and NR-nitrate. Higher dust and sea salt concentrations are likely due to contributions from the Salton Sea[10-12] as well as desert regions[13] located to the northeast of San Diego. For EAS back-trajectories, the NR-organics and NR-nitrate were higher than CNW but not significant. However, NR-nitrate and NR-organics show strong correlations (r = 0.90 at Mt. Soledad and r=0.84 at Scripps Pier, Table S3), suggesting that NR-organics likely share sources with NR-nitrate, possibly contributing to organonitrate formation. The possible upwind influence from near Riverside has been reported to bring both agricultural emissions and mixtures of vehicular (fossil fuel) and residential burning (biomass) combustion sources.[14-16]

To evaluate the regional impact of the different source regions associated with the back-trajectory clusters, we identified eBC, O_3, CO, NO_2, and NO measurements at Air Pollution Control District (APCD) monitoring sites for comparison to EPCAPE. These measurements were available during EPCAPE from eight APCD monitoring sites in San Diego[17] and were compared for the five different back-trajectory clusters identified during EPCAPE at Scripps Pier (Fig. S5). O_3 concentrations for LALB and CNW back-trajectories were generally higher and up to twice as high as those observed for EAS and SOU back-trajectories, indicating that the LALB region remains a large source of O_3 for San Diego[18]. The concentrations of NO_2 were significantly higher for LALB, EAS, and SOU back-trajectories

compared to the CNW. NO_2 concentrations were the highest for EAS back-trajectories at APCD sites, but there were no significant differences in NO concentrations.

rBC and CO are emitted from the incomplete combustion of hydrocarbon-containing fuels[19] and showed significant increases for LALB back-trajectories, with rBC concentrations reaching up to 3.8 times higher than for the EPCAPE average. eBC mass concentrations measured at the inland monitoring stations have an average of 0.26-0.47 μg m^{-3} for CNW back-trajectories, which is less than two times higher than eBC measurements at Scripps Pier (0.15 ± 0.14 μg m^{-3}), consistent with the contributions of additional inland sources. The top and bottom 3% of eBC concentrations were excluded to remove short concentration spikes and negative values. Consistent with prior comparisons [20-22], the uncertainties in the mass absorption coefficient at different locations and in the filter and aerosol loading correction factors, especially at low ambient concentrations typically observed during the campaign, result in a bias between eBC and rBC, often with eBC having higher concentrations than rBC. The inland APCD also have more local BC sources making those values higher than Scripps Pier. At APCD sites in San Diego, eBC concentrations increased by approximately three times for LALB back-trajectories compared to the EPCAPE average and the increase was the most significant at the APCD DVN site near the U.S-Mexico border (Table S4 and Fig. S6). For SOU back-trajectories, CO concentrations were higher at the APCD SAY site near the U.S-Mexico border area compared to at Scripps Pier (Fig. S5), consistent with the transport of rBC and CO from Mexico causing a south-to-north gradient in concentration. This transport of rBC and CO emitted from Mexico to the San Diego area has been suggested by forward trajectory analysis during the Cal-Mex 2010 campaign.[23] In addition to eBC and CO, NO concentration for SOU back-trajectories was significantly higher at the APCD DVN site compared to CNW back-trajectories but differences in NO_2 concentration were not significant (Fig. S5). The high NO associated with SOU back-trajectories likely reflects elevated concentrations from vehicle sources[24, 25] in the Mexico border region.

Text S2. Cosine similarity

Cosine similarity provides a metric for quantifying the relative similarity of two vectors and is used to show the degree to which the mass spectra are similar for each site, between sites, and between different back-trajectory clusters.[26-28] It measures the cosine of the angle between two vectors and is calculated by using the equation below:

$$cosine\ similarity\ = \frac{A\ \cdot B}{\|A\|\|B\|}$$

where each vector A and B are corresponding to the mass spectrum of interest and each reference mass spectrum in the selected database, respectively. $\|A\|$ and $\|B\|$ denote the magnitudes of vectors A and B, and $A{\cdot}B$ indicate the dot product of A and B. The possible range of cosine similarity is from 0 to 1, and the higher the cosine similarity value means the higher the similarity between the mass spectra. This information has been added to the text as follows:

For the back-trajectories, the median cosine similarities for the site-to-site comparison were >0.96 for CNW, LALB, SOU, and EAS. MWE back-trajectories show the lowest median cosine similarity at 0.66 for Scripps (Table S1). Since cosine similarities characterize only the relative similarity of spectra, the median cosine similarities are provided separately for each site for comparison to the values between the sites. For example, the CNW back-trajectories at each site have cosine similarities of 0.98 at Mt. Soledad and 0.93 at Scripps Pier, generally indicating little variability at each site. The site-to-site comparison has a median cosine similarity of 0.98, showing that the variability between sites is also low during CNW back-trajectories. The median standard deviation to mean ratios for CNW, LALB, EAS, and SOU back-trajectories range from 0.33 to 0.48 at Mt Soledad, which are much lower than the range of 0.65 to 1.27 at Scripps Pier due to the higher detection limit and lower resolution of the ACSM compared to the HR-ToF-AMS. MWE back-trajectories had the lowest NR-organic concentrations, which resulted in the highest standard deviation to mean ratios of 1.06 at Mt. Soledad and 2.33 at Scripps Pier.
These 12-month average values are comparable to the cosine similarities of >0.9 that were considered similar by past studies that compared individual measured spectra to positive matrix factorization (PMF) factors[27, 28]. For example, the top 10 cosine similarity values for m/z 1-200 were >0.91 for PMF factors from a month of measurements at Montseny, Spain (DAURE campaign) compared to PMF factors from a separate campaign.[28] In addition, cosine

similarities for PMF factors were above 0.90 for 3-weeks of measurements in Pittsburgh, Pennsylvania, compared to the same database for m/z 1-268.[27]

Text S3. Variability in wind directions and wind speed.

Wind speeds exceed 0.5 m/s for approximately 73–74% of the time over 12 months at both Scripps Pier and Mt. Soledad during the EPCAPE period. Daytime wind direction had lower standard deviation (Day: 27-34 deg, Night: 66-68 deg) as seen in Fig. S7, bringing the air more consistently from offshore. Marine sources are more regionally homogeneous than urban sources, leading to the stronger correlation in concentration of NR-nitrate, NR-organic, and rBC between two sites for sea breezes compared to land breezes (Table S5).

For CNW back-trajectories, the correlations for NR-nitrate, NR-organic, and rBC between the two sites were stronger for sea breeze ($0.37 < r < 0.81$) than for land breeze ($0.26 < r < 0.76$) (Fig. S8 and Table S5). The higher correlations during sea breezes were most likely caused by the higher wind speed with more consistent direction, as well as the lower contributions of the inland sources during sea breezes than during land breezes. The CNW back-trajectories show weaker 24-hr Pearson correlation between two sites compared to the all back-trajectories (Fig. 4) likely due to the lower mass concentrations influenced by upwind regions

Text S4. Lagged correlation

A lagged correlation between Scripps Pier and Mt. Soledad were analyzed for NR-organics, NR-nitrate, NSS-sulfate, rBC, and NR-ammonium by shifting the series of mass concentration at Mt. Soledad for -2 to 2 hrs. The lagged correlation has been analyzed with 30-min average concentration. As seen in Table S6, lagged correlation did not improve the correlation coefficient between two sites.

Tables

Table S1. Cosine similarities of 3-hour average NR-organics m/z spectra within the back-trajectory categories for Mt. Soledad, Scripps Pier, and the site-to-site comparison. Measurements with particle concentrations exceeding 8000 cm^{-3} are removed.

		25th percentile	median	75th percentile
Mt. Soledad	CNW	0.95	0.98	0.99
	LALB	0.98	0.99	1.00
	SOU	0.90	0.96	0.99
	EAS	0.99	0.99	1.00
	MWE	0.84	0.93	0.97
Scripps Pier	CNW	0.82	0.93	0.97
	LALB	0.94	0.98	0.99
	SOU	0.76	0.88	0.95
	EAS	0.95	0.98	0.99
	MWE	0.44	0.66	0.81
Site-to-Site	CNW	0.92	0.98	0.99
	LALB	0.96	0.97	0.99
	SOU	0.88	0.96	0.98
	EAS	0.93	0.96	0.98
	MWE	0.57	0.77	0.89

Table S2. The average mass concentration (standard deviation, µg m^{-3}) of (a) NR-organics, (b) NR-nitrate, (c) rBC and eBC, (d) NSS-sulfate, (e) NR-ammonium, (f) NSS-chloride, (g) Sea salt, and (h) Dust concentrations for EPCAPE average and each clustered back trajectory. Measurements with particle concentrations exceeding 8000 cm^{-3} are removed. The percentage differences from EPCAPE average are given for each back trajectory in red.

Aerosol composition	Site	EPCAPE Average	CNW	LALB	SOU	EAS	MWE
(a) NR-organics	Scripps Pier	1.83 (1.92)	1.58 (1.73) -13%	3.15 (2.50) 73%	1.76 (1.54) -4%	1.77 (1.17) -3%	0.22 (0.19) -88%
	Mt. Soledad	1.96 (2.10)	1.79 (2.20) -9%	2.94 (2.07) 50%	1.90 (1.36) -3%	2.01 (1.11) 3%	0.19 (0.13) -90%
(b) NR-nitrate	Scripps Pier	0.31 (0.58)	0.22 (0.42) -28%	0.72 (0.90) 134%	0.36 (0.88) 16%	0.25 (0.21) -19%	0.03 (0.03) -90%
	Mt. Soledad	0.30 (0.40)	0.24 (0.26) -22%	0.63 (0.94) 108%	0.32 (0.33) 7%	0.29 (0.25) -4%	0.05 (0.01) -83%
(c) Black Carbon	Scripps Pier (rBC)	0.029 (0.036)	0.022 (0.024) -23%	0.051 (0.057) 74%	0.030 (0.027) 3%	0.048 (0.041) 65%	0.002 (0.031) -95%
	Mt. Soledad (rBC)	0.060 (0.081)	0.044 (0.050) -28%	0.104 (0.122) 72%	0.072 (0.080) 19%	0.100 (0.116) 65%	0.009 (0.014) -85%
(d) NSS-sulfate	Scripps Pier	0.75 (0.67)	0.85 (0.70) 14%	0.74 (0.61) -1%	0.53 (0.59) -30%	0.20 (0.14) -73%	0.19 (0.15) -75%
	Mt. Soledad	0.80 (0.69)	0.94 (0.72) 18%	0.66 (0.55) -17%	0.48 (0.59) -40%	0.25 (0.09) -69%	0.16 (0.09) -80%
(e) NR-ammonium	Scripps Pier	0.42 (0.43)	0.43 (0.40) 3%	0.57 (0.56) 38%	0.33 (0.39) -20%	0.14 (0.13) -67%	0.07 (0.08) -84%
	Mt. Soledad	0.32 (0.27)	0.34 (0.27) 7%	0.39 (0.31) 21%	0.24 (0.22) -24%	0.14 (0.07) -58%	0.05 (0.02) -84%
(f) NSS-chloride	Scripps Pier	0.055 (0.065)	0.050 (0.052) -10%	0.090 (0.104) 63%	0.046 (0.047) -17%	0.046 (0.050) -17%	0.030 (0.018) -46%
	Mt. Soledad	0.036 (0.036)	0.034 (0.032) -6%	0.050 (0.047) 39%	0.037 (0.031) 4%	0.033 (0.038) -7%	0.007 (0.006) -79%
(g) Sea salt	Scripps Pier	0.087 (0.102)	0.058 (0.050) -33%	0.067 (0.040) -23%	0.050 (0.005) -42%	0.259 (0.005) 198%	0.499 (0.000) 476%
	Mt. Soledad	0.086 (0.089)	0.071 (0.043) -18%	0.068 (0.036) -21%	0.058 (0.000) -33%	0.299 (0.207) 247%	0.084 (0.000) -2%
(h) Dust	Scripps Pier	0.067 (0.071)	0.049 (0.039) -26%	0.059 (0.020) -12%	0.023 (0.008) -65%	0.281 (0.077) 323%	0.124 (0.000) 87%
	Mt. Soledad	0.122 (0.159)	0.083 (0.054) -32%	0.129 (0.078) 5%	0.041 (0.012) -66%	0.652 (0.172) 434%	0.068 (0.000) -44%

Table S3. Pearson correlation coefficients between chemical compositions for each back-trajectory category at Scripps Pier and Mt. Soledad.

	Site	EPCAPE	CNW	LALB	SOU	EAS	MWE
NR-Organics vs. rBC	Scripps Pier	0.70	0.60	0.74	0.49	0.68	0.37
	Mt. Soledad	0.46	0.45	0.35	0.46	0.81	0.23
NR-Organics vs. NR-Nitrate	Scripps Pier	0.72	0.65	0.69	0.81	0.84	0.31
	Mt. Soledad	0.65	0.60	0.62	0.58	0.90	0.69
NR-Nitrate vs. rBC	Scripps Pier	0.51	0.42	0.57	0.22	0.66	0.60
	Mt. Soledad	0.45	0.44	0.38	0.53	0.70	0.39
NR-Nitrate vs. NR-Ammonium	Scripps Pier	0.51	0.51	0.52	0.54	0.34	0.03
	Mt. Soledad	0.58	0.59	0.78	0.36	0.87	0.33
NR-Organics vs. NSS-Sulfate	Scripps Pier	0.29	0.51	0.08	0.10	0.44	0.33
	Mt. Soledad	0.34	0.54	0.18	0.49	0.10	0.30
NR-Ammonium vs. NSS-Sulfate	Scripps Pier	0.59	0.64	0.49	0.48	0.26	0.13
	Mt. Soledad	0.84	0.92	0.64	0.88	0.27	0.86

Table S4. Locations of the San Diego Air Pollution Control District (APCD) sites in Fig. 1 (e) and their available measurements for comparison to Scripps Pier and Mt. Soledad.[17]

Site ID	Site name	Longitude/Latitude	Measurement
	Scripps Pier (this study)	(-117.2571, 32.8869)	rBC, eBC, CO, O_3
	Mt. Soledad (this study)	(-117.2494, 32.8393)	rBC, O_3
MRT	Marine terminal	(-117.161, 32.7007)	eBC
CBA	Caltrans at Boston Ave.	(-117.1316, 32.694)	eBC
OVB	Oceanview Blvd	(-117.1196, 32.7021)	eBC
CCP	Chicano Park	(-117.1443, 32.7009)	eBC
CMP	Camp Pendleton	(-117.3962, 33.2170)	NO, NO_2, O_3
CVA	Chula Vista	(-117.0591, 32.6312)	NO_2, O_3
DVN	Otay Mesa – Donovan	(-116.9213, 32.5782)	NO, NO_2
KVR	Kearny Villa Rd.	(-117.1240, 32.8457)	NO_2, O_3
LES	Lexington Elementary School	(-116.9443, 32.7896)	NO_2, O_3
RCD	Rancho Carmel Dr.	(-117.0822, 32.9854)	NO_2, CO
SAY	San Ysidro	(-117.0473, 32.5528)	NO_2, CO
SES	Downtown-Sherman Elementary School	(-117.1427, 32.7102)	eBC, NO_2, O_3

Table S5. Slope of the regression line and Pearson's correlation coefficients (R) of 30-minute averaged mass concentration for NR-organics, NR-nitrate, rBC, NSS-sulfate, and NR-ammonium concentrations at Mt. Soledad compared to Scripps Pier for all back-trajectories from Figure S9. Measurements with particle concentrations exceeding 8000 cm^{-3} are removed. The percentage differences in mean mass concentration of NR-organics, NR-nitrate, rBC, NSS-sulfate, and NR-ammonium for land-sea breeze difference between the land breeze (1658 hrs) and the sea breeze (2042 hrs) relative to the EPCAPE average at each site. Asterisks (*) denote significant differences based on t-test (p-value < 0.05). Mean mass concentrations are given in the parenthesis for each site (Sea breeze / Land breeze).

	Regression slope			Correlation coefficient (R)			Land-sea breeze difference	
	24-hr	Sea breeze	Land breeze	24-hr	Sea breeze	Land breeze	Scripps Pier	Mt. Soledad
NR-organics	0.77	0.82	0.86	0.88	0.81	0.76	5%<br>(2.00 / 2.10)	-2%<br>(2.15 / 2.11)
NR-nitrate	0.61	0.77	0.79	0.51	0.59	0.54	24%*<br>(0.28 / 0.35)	30%*<br>(0.32 / 0.40)
rBC	0.47	0.51	0.37	0.65	0.76	0.49	59%*<br>(0.032 / 0.049)	20%*<br>(0.060 / 0.072)
NSS-sulfate	0.87	0.97	0.77	0.71	0.71	0.68	-15%<br>(0.83 / 0.72)	-3%<br>(0.33 / 0.35)
NR-ammonium	1.08	1.11	1.06	0.52	0.37	0.26	17%<br>(0.40 / 0.47)	5%<br>(0.33 / 0.35)

Table S6. Lagged correlation coefficients for 30-minute average mass concentration of NR-organics, NR-nitrate, rBC, NSS-sulfate, and NR-ammonium between Scripps Pier and Mt. Soledad for sea breeze and land breeze conditions for CNW back-trajectories. Time lag is applied to the mass concentration at Mt. Soledad, meaning that when the time lag is 2 hours, the concentration at Mt. Soledad is shifted by 2 hours.

Time lag (hr)	Correlation coefficient (Sea breeze / Land breeze)				
	NR-organics	NR-nitrate	rBC	NSS-sulfate	NR-ammonium
-2	0.82 / 0.64	0.58 / 0.47	0.56 / 0.48	0.71 / 0.57	0.47 / 0.12
-1.5	0.83 / 0.56	0.61 / 0.55	0.58 / 0.49	0.71 / 0.56	0.50 / 0.12
-1	0.85 / 0.65	0.64 / 0.56	0.60 / 0.49	0.71 / 0.57	0.48 / 0.10
-0.5	0.86 / 0.71	0.68 / 0.61	0.60 / 0.55	0.71 / 0.59	0.49 / 0.14
0	0.86 / 0.70	0.69 / 0.61	0.61 / 0.54	0.70 / 0.61	0.48 / 0.29
0.5	0.86 / 0.72	0.67 / 0.63	0.61 / 0.53	0.71 / 0.58	0.50 / 0.15
1	0.87 / 0.72	0.65 / 0.60	0.59 / 0.55	0.69 / 0.59	0.47 / 0.14
1.5	0.86 / 0.66	0.62 / 0.56	0.59 / 0.48	0.68 / 0.59	0.45 / 0.14
2	0.85 / 0.74	0.60 / 0.52	0.57 / 0.47	0.67 / 0.60	0.45 / 0.13

Figures

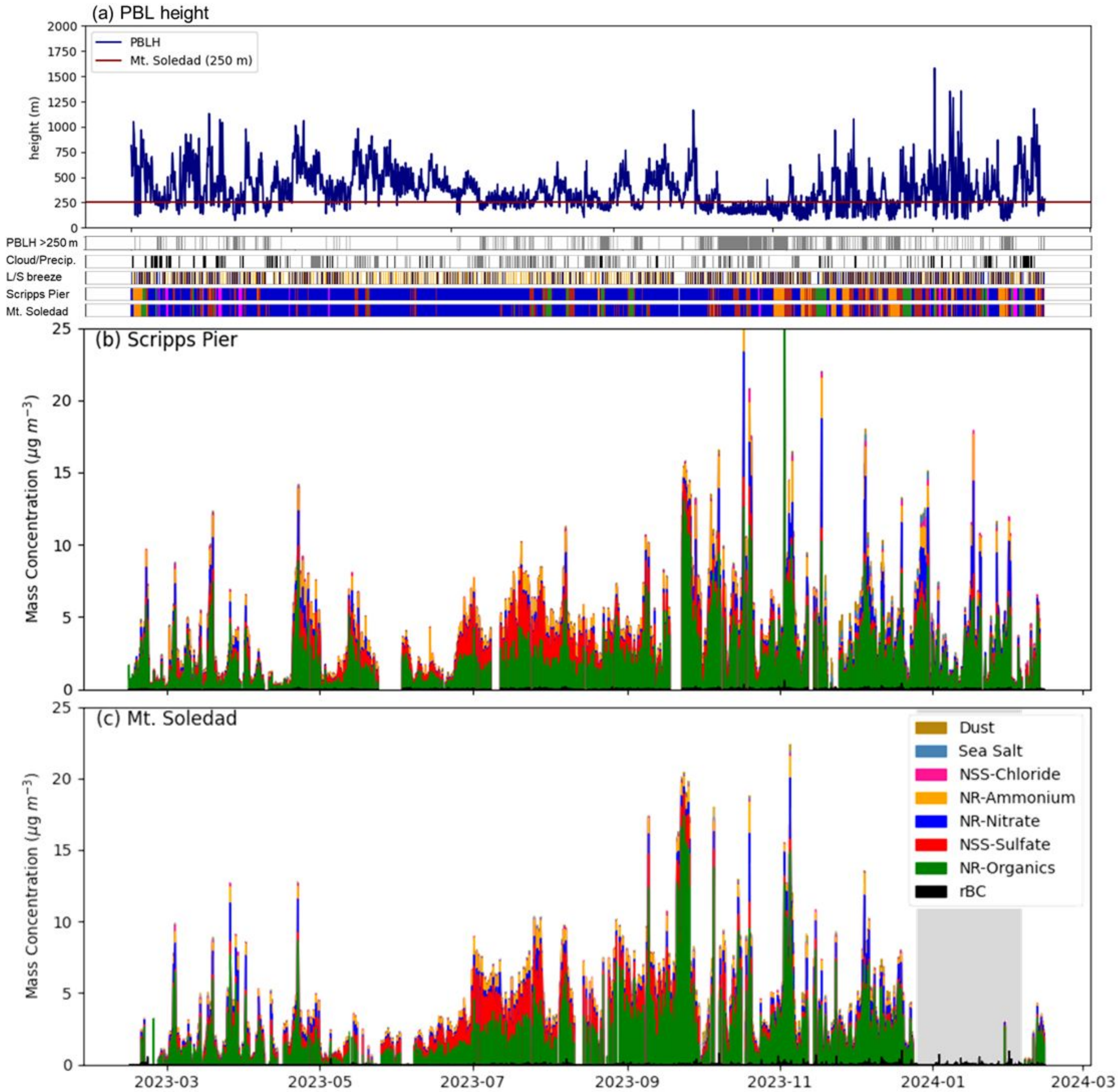

Figure S1. Time series comparing aerosol composition at Scripps Pier and Mt. Soledad using 3-hr average measurements. (a) Planetary Boundary Layer Height (PBLH) estimated using the LightGBM method[29] at Scripps Pier (navy), with the altitude of the Mt. Soledad sampling site shown for reference (red). The first two bar graphs below panel (a) indicate times when the Mt. Soledad site was above the PBLH, followed by times excluded because of clouds at Mt. Soledad (gray) or rain (black). The third bar graph shows times meeting the land (orange) and sea (navy) breeze (L/S Breeze) criteria for 3-hr intervals. The next two color bars indicate back-trajectory cluster classifications (3-hour intervals), including CNW (blue), LALB (red), SOU (green), EAS (orange), and MWE (magenta). Panels (b) and (c) display time series of rBC, NR-organics, NSS-sulfate, NR-nitrate, and NR-ammonium PM1 concentrations at Scripps Pier and Mt. Soledad, including times with rain and clouds at Mt. Soledad but excluding times with number concentrations exceeding 8000 cm^{-3}. The gray-shaded region indicates times when the AMS at Mt. Soledad was offline for maintenance.

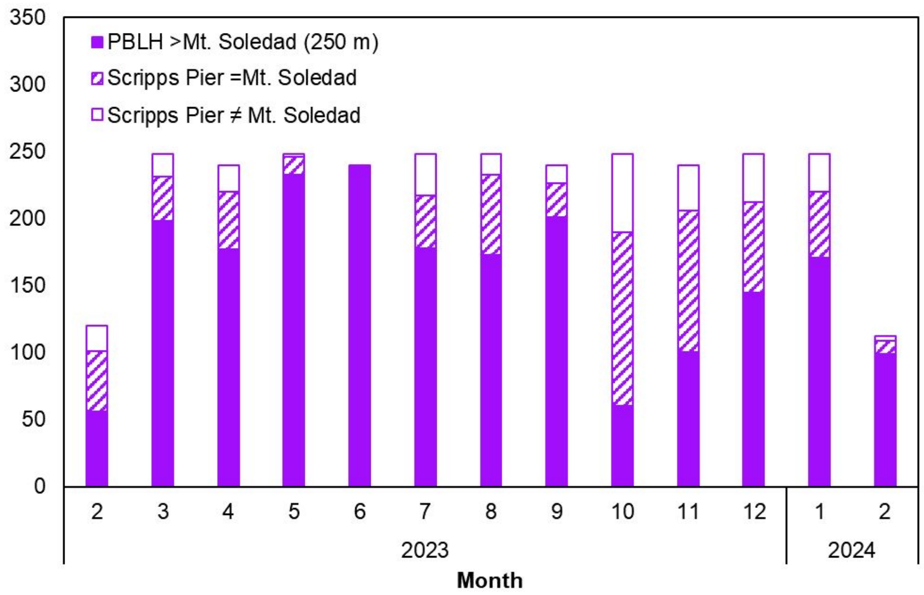

Figure S2. Number of back trajectories when PBLH is above Mt. Soledad (filled), PBLH is below Mt. Soledad but clustered to the same back-trajectory categories as for Scripps Pier (dashed), and PBLH is below Mt. Soledad and clustered to a different back-trajectory category than for Scripps Pier (empty).

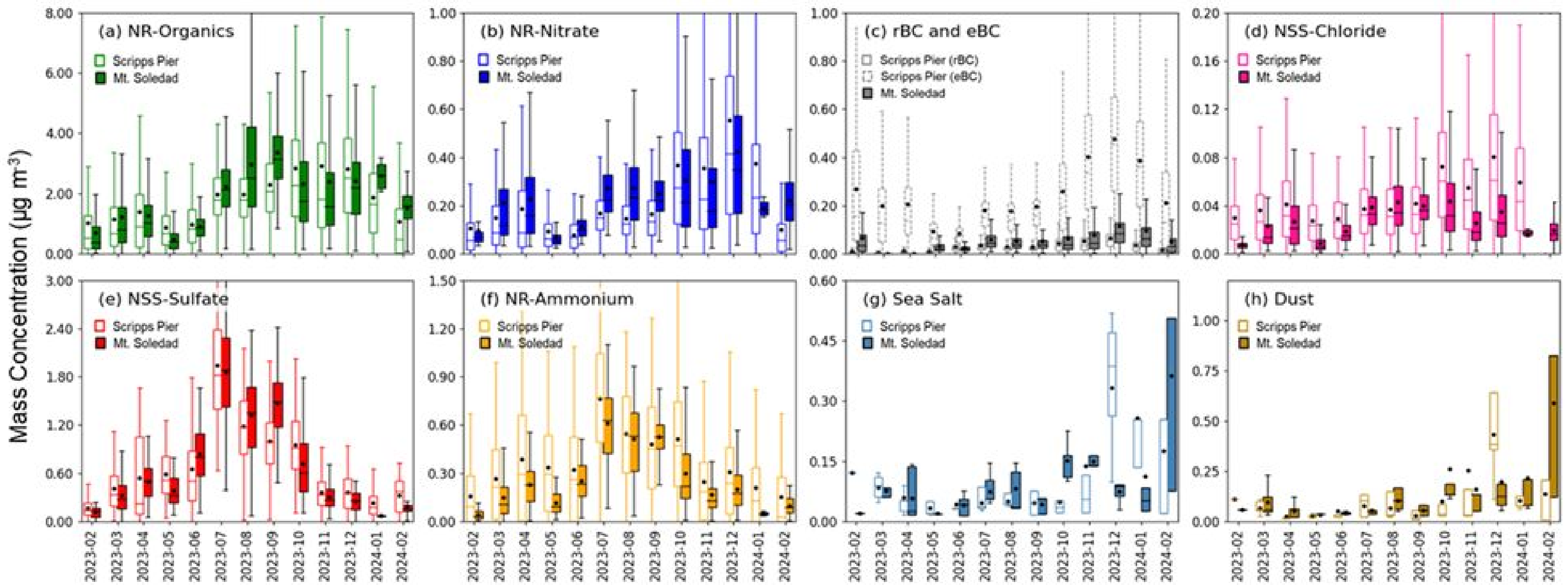

Figure S3. Box plots of 3- hour average mass concentrations (μg m^{-3}) of (a) NR-organics, (b) NR-nitrate, (c) eBC and rBC, (d) NSS-chloride (e) NSS-sulfate, (f) NR-ammonium, (g) sea salt, and (h) dust in PM1 for each month at Scripps Pier (empty) and Mt. Soledad (filled) for all back trajectories, excluding times with rain and clouds at Mt. Soledad and number concentrations exceeding 8000 cm^{-3}.. The symbols represent the monthly means. The box represents the median, the upper (75th percentile) and lower (25th percentile) quartiles. The caps mark the maximum and minimum non-outlier values. Measurements with particle concentrations exceeding 8000 cm^{-3} are removed.

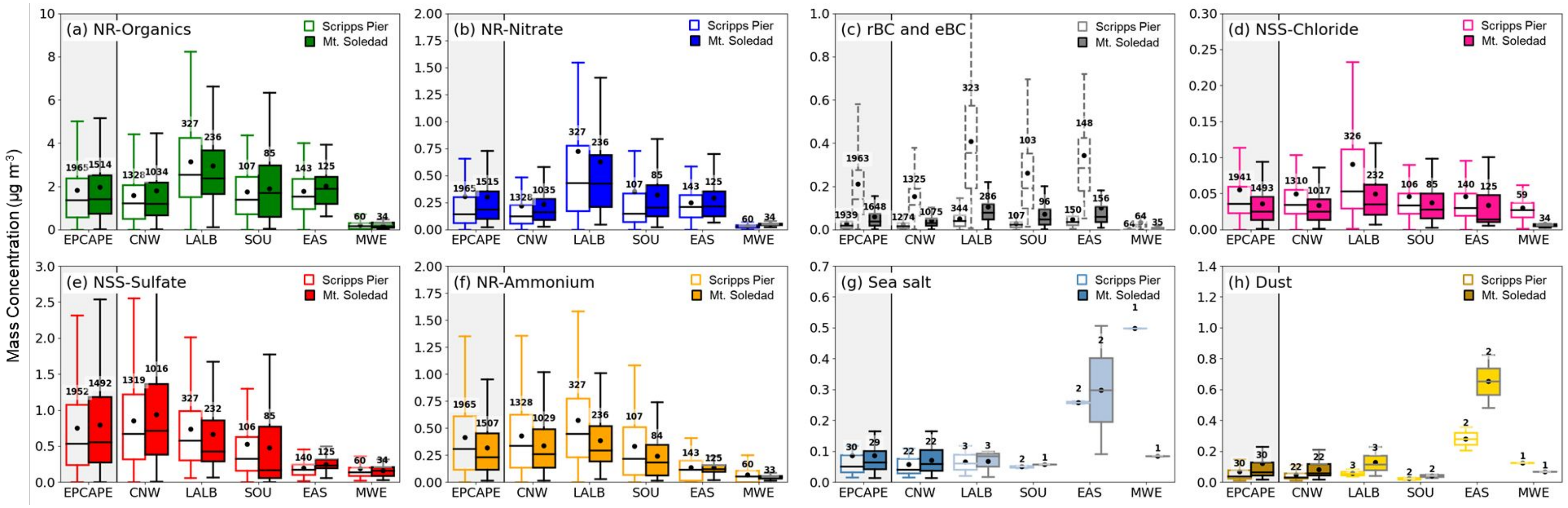

Figure S4. Box plots of 3-hour average mass concentrations (μg m^{-3}) for (a) NR-organics, (b) NR-nitrate, (c) rBC and eBC, (d) NSS-chloride, (e) NSS-sulfate, (f) NR-ammonium, (g) sea salt, and (h) Dust in PM1 at Mt. Soledad (filled) and Scripps Pier (hollow), categorized by the five identified air mass origins when the air mass origin of Mt. Soledad was the same as Scripps Pier (91% of time, Fig S1), excluding times with rain and clouds at Mt. Soledad and number concentrations exceeding 8000 cm^{-3}.. Numbers above box are the number of 3-hourly samples for each category. For Scripps Pier, eBC concentrations are shown as dashed lined box plots with rBC in (c). The symbols represent the mean. The box represents the median, the upper (75th percentile) and lower (25th percentile) quartiles. The caps mark the maximum and minimum non-outlier values.

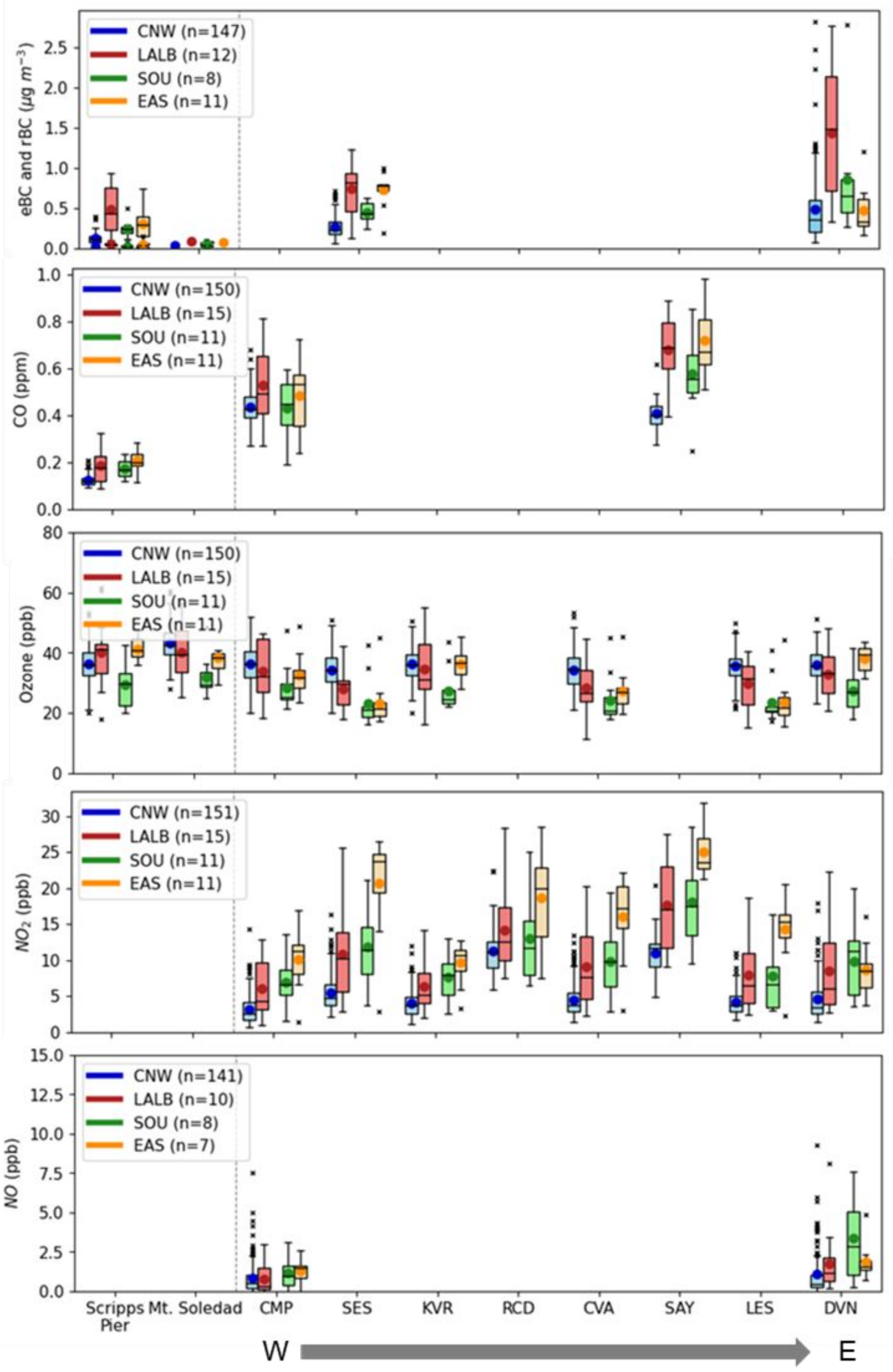

Figure S5. Box plots of daily average mass concentrations of eBC and rBC (μg m^{-3}), and mixing ratios of CO (ppm), ozone (ppb), NO_2 (ppb), and NO (ppb) at the selected APCD sites (see Table S4) with Scripps pier and Mt. Soledad, categorized by the four identified air mass origins at Scripps Pier (Coastal NW, LA-LB, Southerly, and Easterly). These selected stations are sites with more than two of measurement among eBC, rBC, CO, O_3, NO_2, and NO. For Scripps Pier, rBC concentrations are shown as dashed lined box plots with eBC. The symbols represent average values. The box represents the median, the upper (75th percentile) and lower (25th percentile) quartiles. The caps mark the maximum and minimum non-outlier values.

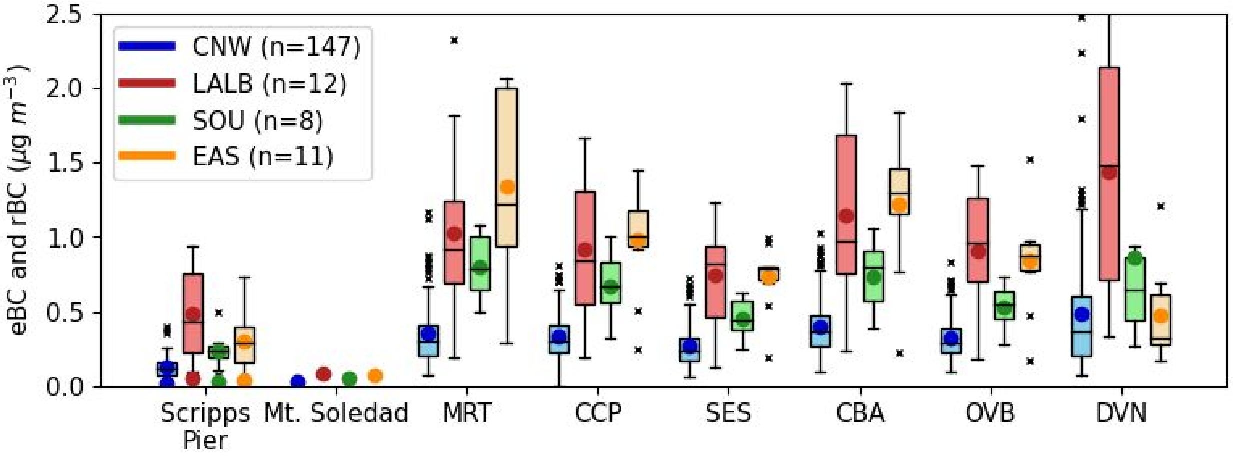

Figure S6. Box plots of daily average mass concentrations of eBC (μg m^{-3}) measured by aethalometer at six APCD sites (see Table S1 and Fig. S5) and by SP2 at Scripps Pier and Mt. Soledad, categorized by the four identified air mass origins at Scripps Pier (CNW, LALB, SOU, EAS). The symbols represent averaged values. For Scripps Pier and Mt. Soledad, the averaged mass concentration of rBC are shown. Measurements at Scripps Pier and Mt. Soledad with particle concentrations exceeding 8000 cm^{-3} are removed.

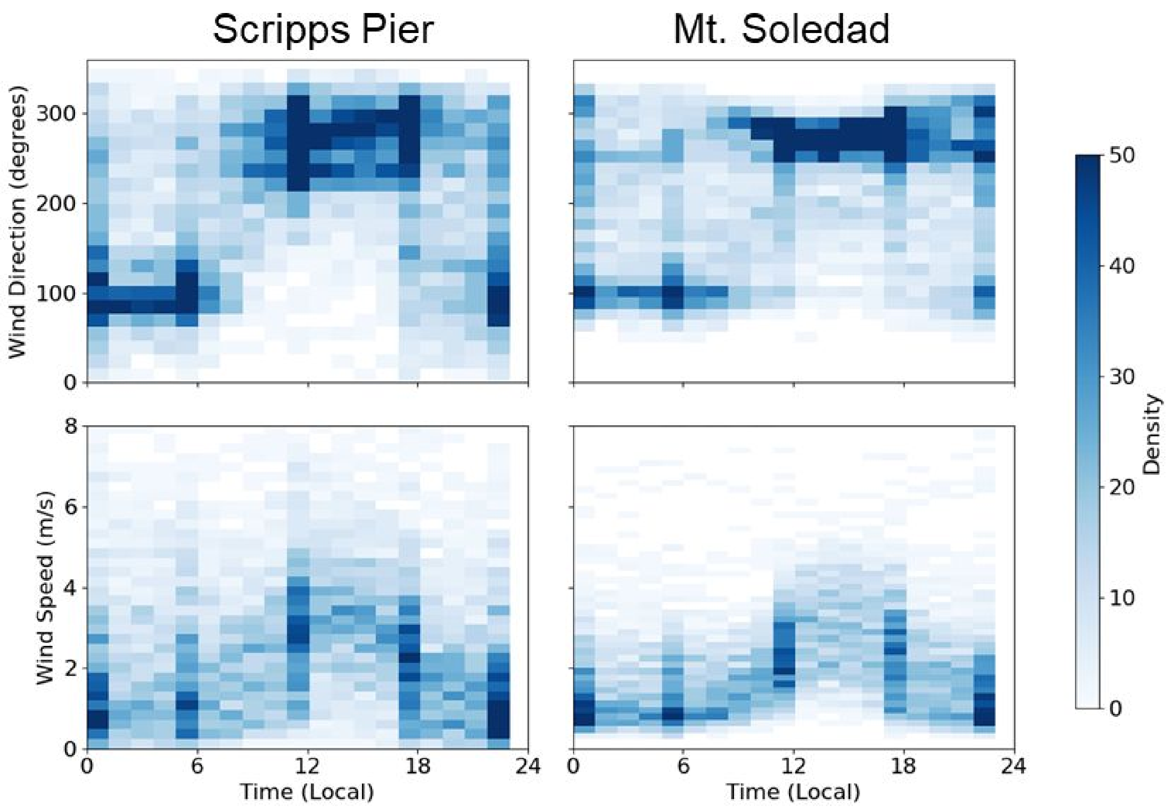

Figure S7. Density plot of wind directions and wind speeds along with the hour of the day at Scripps Pier (left) and Mt. Soledad (right) for all back-trajectories.

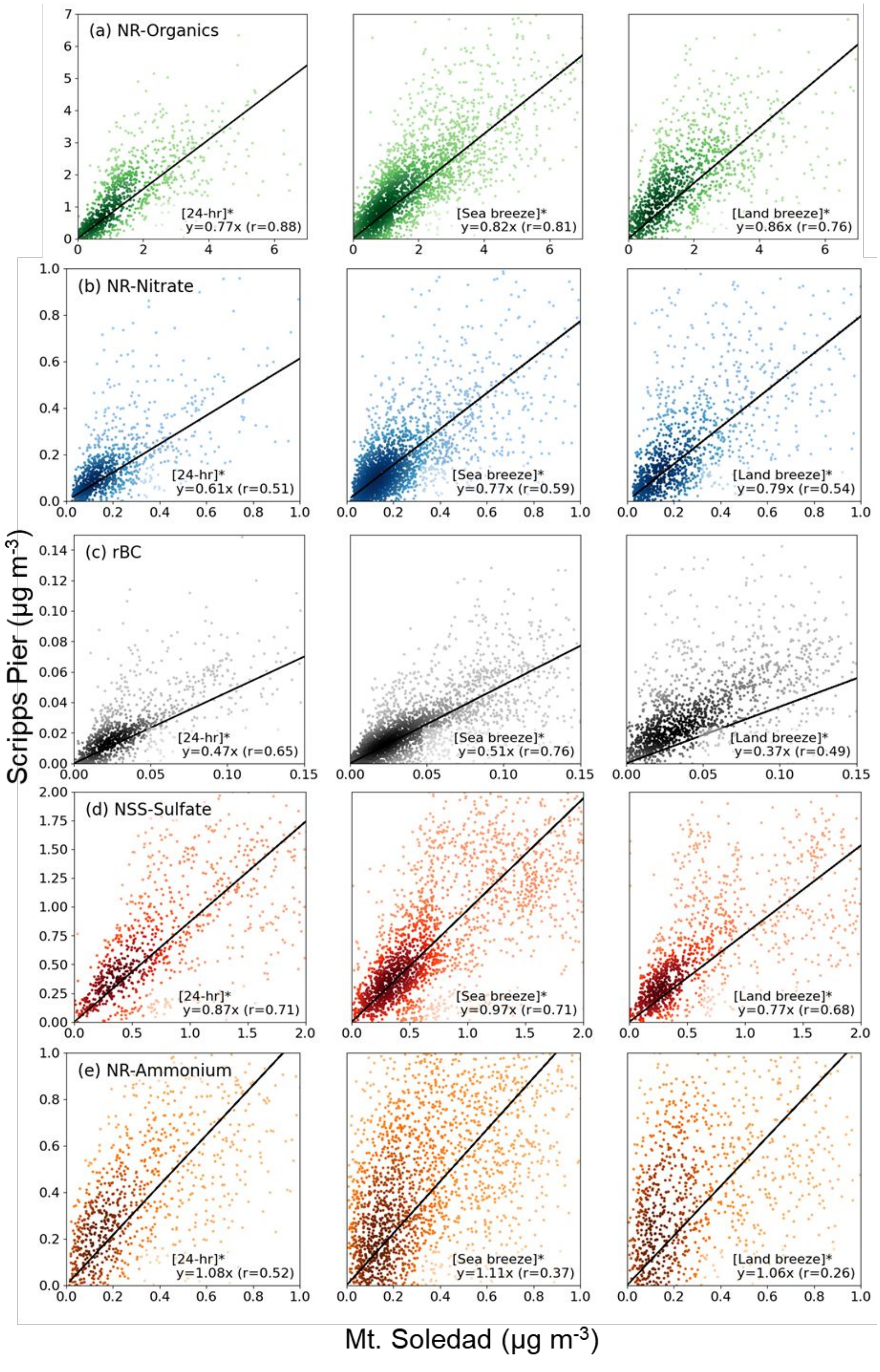

Figure S8. Density scatter plots of 30-minute averaged mass concentrations (µg m^{-3}) for 24-hour periods, sea breeze, and land breezes at Scripps Pier to Mt. Soledad for (a) NR-organics, (b) NR-nitrate, (c) rBC, (d) NSS-sulfate, and (e) NR-ammonium during EPCAPE for all back-trajectories, excluding times with rain and clouds at Mt. Soledad and number concentrations exceeding 8000 cm^{-3}.

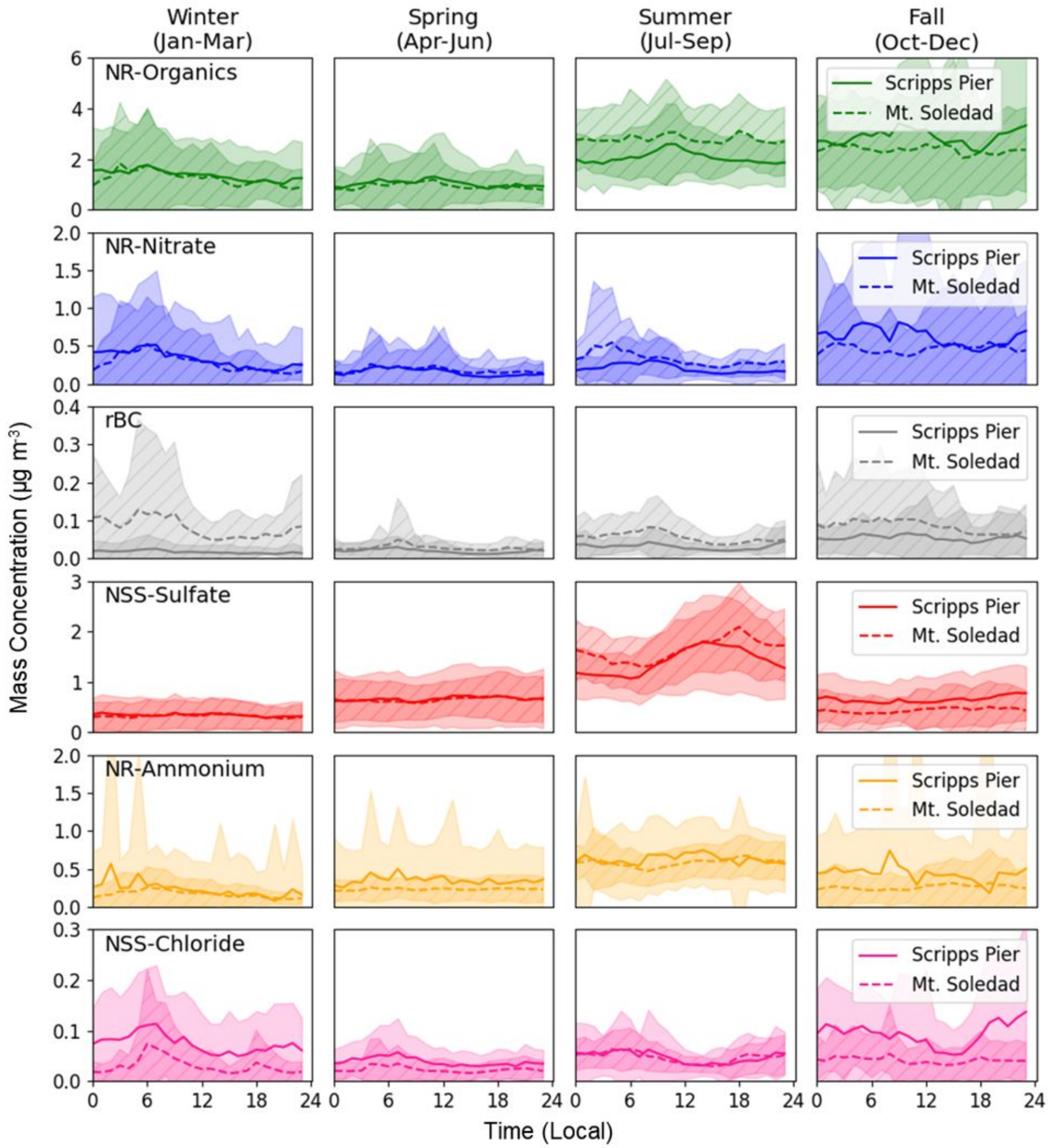

Figure S9. Diurnal patterns in NR-organics, NR-nitrate, rBC, NSS-sulfate, NR-ammonium, and NSS-chloride at Scripps Pier (solid line) and Mt. Soledad (dashed line) for all back-trajectories, excluding times with rain and clouds at Mt. Soledad and number concentrations exceeding 8000 cm^{-3}. The shaded area represents the standard deviation at Scripps Pier, while the dashed shaded area represents the standard deviation at Mt. Soledad.

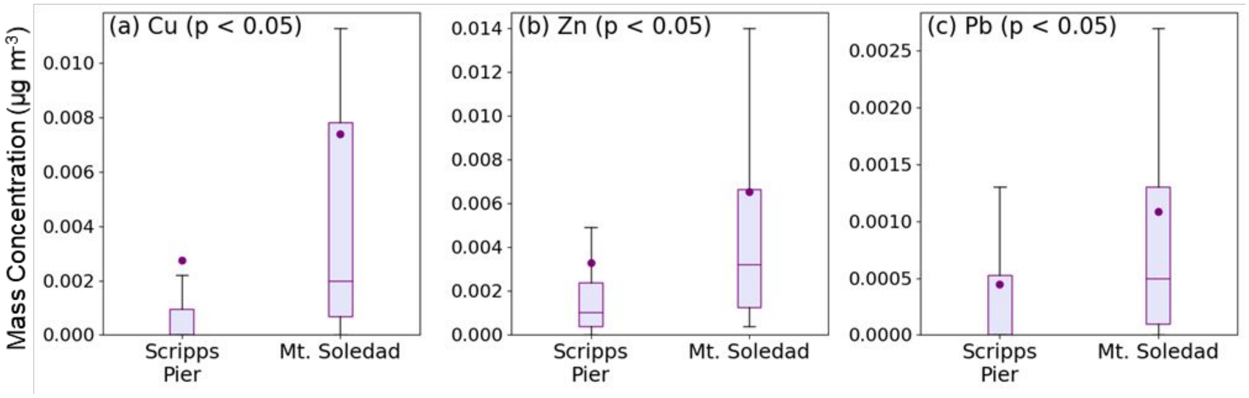

Figure S10. Box plots of Cu, Zn, and Pb concentrations at Scripps Pier and Mt. Soledad for all back-trajectories, excluding times with rain and clouds at Mt. Soledad and number concentrations exceeding 8000 cm^{-3}.. The p-values are derived from t-tests, indicating the significance of differences in mass concentrations between the two sites. The symbols represent mean values. The box represents the median, the upper (75th percentile) and lower (25th percentile) quartiles. The caps mark the maximum and minimum non-outlier values.

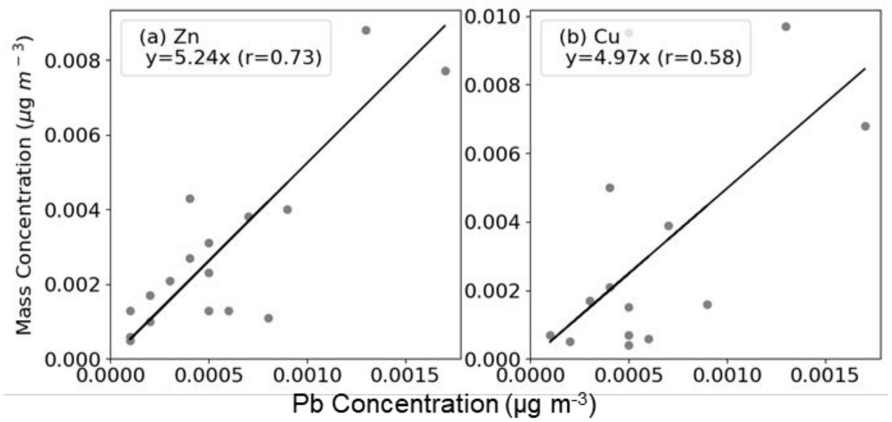

Figure S11. The lower panel shows the correlation of Cu and Zn to with the Pearson's correlation coefficient (r).

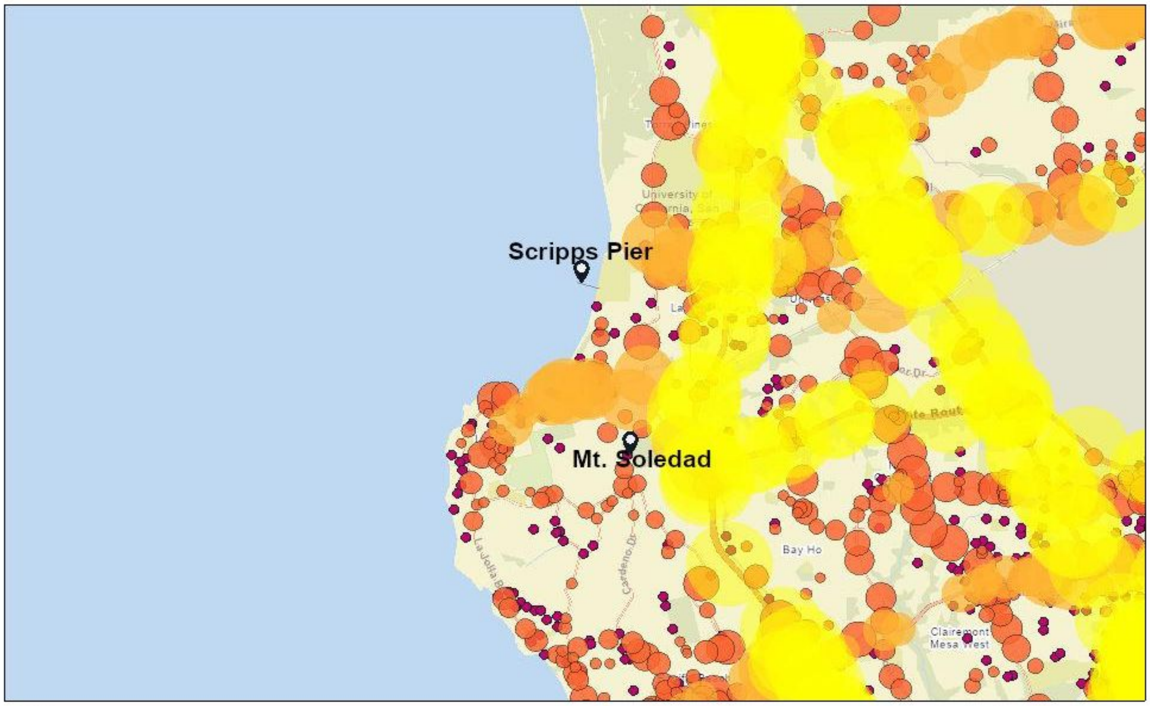

Figure S12. The Esri Traffic Counts data tracks the average daily traffic volume (https://maps.arcgis.com/apps/mapviewer/index.html?webmap=ced1855778634da6b72516ec2f33b219), indicating the number of vehicles that cross a specific point on a street based on traffic counts from across the United States for the most recent available calendar year (2022). Yellow circles represent vehicle counts exceeding 60,000, orange indicates counts between 30,000 and 60,000, dark orange denotes counts between 5,000 and 30,000, and red signifies counts below 5,000.

References

(1) Houston, D.; Li, W.; Wu, J. Disparities in Exposure to Automobile and Truck Traffic and Vehicle Emissions Near the Los Angeles–Long Beach Port Complex. *American Journal of Public Health* **2013**, *104* (1), 156-164. DOI: 10.2105/AJPH.2012.301120 (acccessed 2024/11/27).

(2) Hu, S.; Paulson, S. E.; Fruin, S.; Kozawa, K.; Mara, S.; Winer, A. M. Observation of elevated air pollutant concentrations in a residential neighborhood of Los Angeles California using a mobile platform. *Atmospheric Environment* **2012**, *51*, 311-319. DOI: https://doi.org/10.1016/j.atmosenv.2011.12.055.

(3) Ault, A. P.; Moore, M. J.; Furutani, H.; Prather, K. A. Impact of emissions from the Los Angeles port region on San Diego air quality during regional transport events. *Environmental science & technology* **2009**, *43* (10), 3500-3506. DOI: 10.1021/es8018918.

(4) Gaston, C. J.; Quinn, P. K.; Bates, T. S.; Gilman, J. B.; Bon, D. M.; Kuster, W. C.; Prather, K. A. The impact of shipping, agricultural, and urban emissions on single particle chemistry observed aboard the R/V Atlantis during CalNex. *Journal of Geophysical Research: Atmospheres* **2013**, *118* (10), 5003-5017. DOI: 10.1002/jgrd.50427.

(5) Liu, S.; Day, D.; Shields, J.; Russell, L. Ozone-driven daytime formation of secondary organic aerosol containing carboxylic acid groups and alkane groups. *Atmospheric Chemistry and Physics* **2011**, *11* (16), 8321-8341. DOI: 10.5194/acp-11-8321-2011.

(6) Hawkins, L. N.; Russell, L. M. Oxidation of ketone groups in transported biomass burning aerosol from the 2008 Northern California Lightning Series fires. *Atmospheric Environment* **2010**, *44* (34), 4142-4154. DOI: 10.1016/j.atmosenv.2010.07.036.

(7) Lee, A. K.; Chen, C.-L.; Liu, J.; Price, D. J.; Betha, R.; Russell, L. M.; Zhang, X.; Cappa, C. D. Formation of secondary organic aerosol coating on black carbon particles near vehicular emissions. *Atmospheric Chemistry and Physics* **2017**, *17* (24), 15055-15067. DOI: 10.5194/acp-17-15055-2017.

(8) Cui, S.; Huang, D. D.; Wu, Y.; Wang, J.; Shen, F.; Xian, J.; Zhang, Y.; Wang, H.; Huang, C.; Liao, H. Chemical properties, sources and size-resolved hygroscopicity of submicron black carbon-containing aerosols in urban Shanghai. *Atmospheric Chemistry and Physics Discussions* **2022**, *2022*, 1-58. DOI: 10.5194/acp-22-8073-2022.

(9) Takahama, S.; Johnson, A.; Morales, J. G.; Russell, L. M.; Duran, R.; Rodriguez, G.; Zheng, J.; Zhang, R.; Toom-Sauntry, D.; Leaitch, W. R. Submicron organic aerosol in Tijuana, Mexico, from local and Southern California sources during the CalMex campaign. *Atmospheric Environment* **2013**, *70*, 500-512. DOI: 10.1016/j.atmosenv.2012.07.057.

(10) Frie, A. L.; Garrison, A. C.; Schaefer, M. V.; Bates, S. M.; Botthoff, J.; Maltz, M.; Ying, S. C.; Lyons, T.; Allen, M. F.; Aronson, E. Dust sources in the Salton Sea Basin: A clear case of an anthropogenically impacted dust budget. *Environmental science & technology* **2019**, *53* (16), 9378-9388. DOI: 10.1021/acs.est.9b02137.

(11) Frie, A. L.; Dingle, J. H.; Ying, S. C.; Bahreini, R. The effect of a receding saline lake (the Salton Sea) on airborne particulate matter composition. *Environmental science & technology* **2017**, *51* (15), 8283-8292. DOI: 10.1021/acs.est.7b01773.

(12) King, J.; Etyemezian, V.; Sweeney, M.; Buck, B. J.; Nikolich, G. Dust emission variability at the Salton Sea, California, USA. *Aeolian Research* **2011**, *3* (1), 67-79. DOI: 10.1016/j.aeolia.2011.03.005.

(13) Álvarez, C. A.; Carbajal, N.; Pineda-Martínez, L. F. Dust pollution caused by an extreme Santa Ana wind event. *Natural Hazards* **2022**, *110* (3), 1427-1442. DOI: 10.1007/s11069-021-04996-z.

(14) Day, D. A.; Liu, S.; Russell, L. M.; Ziemann, P. J. Organonitrate group concentrations in submicron particles with high nitrate and organic fractions in coastal southern California. *Atmospheric Environment* **2010**, *44* (16), 1970-1979. DOI: 10.1016/j.atmosenv.2010.02.045.

(15) Docherty, K.; Aiken, A. C.; Huffman, J.; Ulbrich, I.; DeCarlo, P.; Sueper, D.; Worsnop, D.; Snyder, D.; Peltier, R.; Weber, R. The 2005 Study of Organic Aerosols at Riverside (SOAR-1): instrumental intercomparisons and fine particle composition. *Atmospheric Chemistry and Physics* **2011**, *11* (23), 12387-12420. DOI: 10.5194/acp-11-12387-2011.

(16) Docherty, K. S.; Stone, E. A.; Ulbrich, I. M.; DeCarlo, P. F.; Snyder, D. C.; Schauer, J. J.; Peltier, R. E.; Weber, R. J.; Murphy, S. M.; Seinfeld, J. H. Apportionment of primary and secondary organic aerosols in Southern California during the 2005 Study of Organic Aerosols in Riverside (SOAR-1). *Environmental science & technology* **2008**, *42* (20), 7655-7662. DOI: 10.1021/es8008166.

(17) (SDAPCD), S. D. A. P. C. D. *Air quality monitoring data*. https://www.sdapcd.org/content/sdapcd/air-quality.html (accessed 2024 10/15/2024).

(18) Luria, M.; Tanner, R.; Valente, R.; Bairai, S.; Koracin, D.; Gertler, A. Local and transported pollution over San Diego, California. *Atmospheric Environment* **2005**, *39* (36), 6765-6776. DOI: 10.1016/j.atmosenv.2005.07.051.

(19) Wang, Y.; Wang, X.; Kondo, Y.; Kajino, M.; Munger, J. W.; Hao, J. Black carbon and its correlation with trace gases at a rural site in Beijing: Top-down constraints from ambient measurements on bottom-up emissions. *Journal of Geophysical Research: Atmospheres* **2011**, *116* (D24). DOI: 10.1029/2011JD016575.
(20) Laing, J. R.; Jaffe, D. A.; Sedlacek, I. I. I. A. J. Comparison of Filter-based Absorption Measurements of Biomass Burning Aerosol and Background Aerosol at the Mt. Bachelor Observatory. *Aerosol and Air Quality Research* **2020**, *20* (4), 663-678. DOI: 10.4209/aaqr.2019.06.0298.
(21) Chakrabarty, R. K.; Shetty, N. J.; Thind, A. S.; Beeler, P.; Sumlin, B. J.; Zhang, C.; Liu, P.; Idrobo, J. C.; Adachi, K.; Wagner, N. L.; et al. Shortwave absorption by wildfire smoke dominated by dark brown carbon. *Nature Geoscience* **2023**, *16* (8), 683-688. DOI: 10.1038/s41561-023-01237-9.
(22) Tinorua, S.; Denjean, C.; Nabat, P.; Pont, V.; Arnaud, M.; Bourrianne, T.; Dias Alves, M.; Gardrat, E. A 2-year intercomparison of three methods for measuring black carbon concentration at a high-altitude research station in Europe. *Atmos. Meas. Tech.* **2024**, *17* (13), 3897-3915. DOI: 10.5194/amt-17-3897-2024.
(23) Shores, C. A.; Klapmeyer, M. E.; Quadros, M. E.; Marr, L. C. Sources and transport of black carbon at the California–Mexico border. *Atmospheric Environment* **2013**, *70*, 490-499. DOI: 10.1016/j.atmosenv.2012.04.031.
(24) Bishop, G. A.; Stedman, D. H. A decade of on-road emissions measurements. *Environmental Science & Technology* **2008**, *42* (5), 1651-1656. DOI: 10.1021/es702413b.
(25) McDonald, B. C.; Dallmann, T. R.; Martin, E. W.; Harley, R. A. Long-term trends in nitrogen oxide emissions from motor vehicles at national, state, and air basin scales. *Journal of Geophysical Research: Atmospheres* **2012**, *117* (D21). DOI: 10.1029/2012JD018304.
(26) Stein, S. E.; Scott, D. R. Optimization and testing of mass spectral library search algorithms for compound identification. *Journal of the American Society for Mass Spectrometry* **1994**, *5* (9), 859-866. DOI: 10.1016/1044-0305(94)87009-8.
(27) Ulbrich, I.; Canagaratna, M.; Zhang, Q.; Worsnop, D.; Jimenez, J. Interpretation of organic components from Positive Matrix Factorization of aerosol mass spectrometric data. *Atmospheric Chemistry and Physics* **2009**, *9* (9), 2891-2918. DOI: 10.5194/acp-9-2891-2009.
(28) Jeon, S.; Walker, M. J.; Sueper, D. T.; Day, D. A.; Handschy, A. V.; Jimenez, J. L.; Williams, B. J. A searchable database and mass spectral comparison tool for the Aerosol Mass Spectrometer (AMS) and the Aerosol Chemical Speciation Monitor (ACSM). *Atmospheric Measurement Techniques* **2023**, *16* (24), 6075-6095. DOI: 10.5194/amt-16-6075-2023.
(29) Ke, G.; Meng, Q.; Finley, T.; Wang, T.; Chen, W.; Ma, W.; Ye, Q.; Liu, T.-Y. Lightgbm: A highly efficient gradient boosting decision tree. *Advances in neural information processing systems* **2017**, *30*.
